# High-temperature chemical oxidation pathways in lithium-ion batteries: mechanistic insights into ethylene carbonate decomposition

**DOI:** 10.1039/d6sc00426a

**Published:** 2026-03-09

**Authors:** Leon Schmidt, Kie Hankins, Jorge Valenzuela, Rene Windiks, Adrian Lindner, Ruth Witzel, Yuchen Qiu, Edwin Knobbe, Ulrike Krewer

**Affiliations:** a Institute for Applied Materials - Electrochemical Technologies, Karlsruhe Institute of Technology 76131 Karlsruhe Germany ulrike.krewer@kit.edu; b Materials Design SARL 42 Avenue Verdier 92120 Montrouge France; c BMW Group, Battery Cell Competence Center 80788 Munich Germany

## Abstract

A thermal event remains a safety challenge for lithium-ion batteries due to the self-reinforcing nature of the exothermic reactions occurring at elevated temperatures. Higher states of charge have been shown to exacerbate the onset and severity of a thermal event. For cells containing Ni-rich layered oxide-based electrodes, this has been attributed to the increased instability of the material leading to lattice oxygen release. The degradation reactions on the electrode/electrolyte interface triggered by this oxygen remain insufficiently understood. In this study, we investigate high-temperature degradation pathways of ethylene carbonate (EC)-based electrolytes in contact with Ni-rich positive electrode active materials up to 130 °C. By combining *in situ* high-temperature online electrochemical mass spectrometry with post-mortem analyses, we identify and validate key degradation intermediates and products. Two distinct EC oxidation pathways are revealed: one activated at high voltages, and the other one initiated by traces of water impurities. Complementary density functional theory calculations show the reactions are thermodynamically favorable and quantify the heat release associated with each pathway. Both pathways produce significant heat and lead to gassing of CO_2_ and H_2_. These findings suggest a significant contribution of EC to thermal gas evolution and exothermicity under abuse conditions, thereby establishing a mechanistic link between electrolyte chemistry and thermal events. This integrated experimental–computational approach provides critical insights to guide improved electrolyte formulations and predictive thermal models.

## Abstract 2

Under thermal abuse conditions, the interplay of state of charge and trace water impurities drives the degradation of ethylene carbonate-based electrolytes on Ni-rich positive electrodes in lithium-ion batteries.

## Graphical abstract

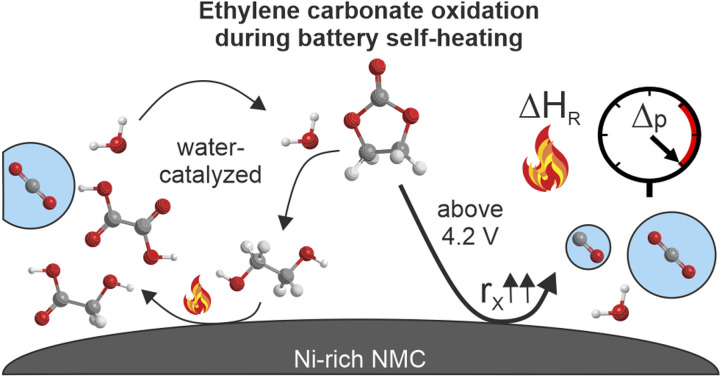

## Introduction

The widespread deployment of lithium-ion batteries (LIBs) in the mobility sector has brought new technical innovations, with safety remaining a major challenge: while the usage under mild conditions is generally considered safe, harsh operating conditions, such as temperatures above 80 °C, increase the probability of cell failure.^1,2^ This can trigger a cascade of exothermic degradation reactions of battery materials, causing self-heating of the battery. In severe cases, this can lead to a thermal event, a rapid uncontrolled series of reactions that causes temperature rises of several hundred °C.^2,3^

Self-heating may commence at temperatures as low as 70–80 °C *via* exothermic reactions involving the decomposition of the Solid Electrolyte Interphase (SEI),^4^ a surface film of lithium salts and polymers initially formed on the negative electrode during first cycling.^5^ The decomposition and reformation reactions of the SEI are exothermic, making this component a significant factor with regards to the overall safety of the cell.^6,7^ Higher temperatures above 130 °C can trigger the breakdown and oxygen release of positive electrodes containing transition metal oxides such as lithium nickel manganese cobalt oxide (NMC) with high nickel content (≥80%).^8^ At such high temperatures, oxygen contributes to the decomposition of the electrolyte, further driving the heat evolution during a thermal event.^2,9,10^

While the macroscopic consequences of a thermal event are well documented, the detailed chemical pathways remain poorly understood. Identifying the degradation pathways and the source of self-heating is crucial for a knowledge-driven design of cells that are safe and chemically stable. Previous studies focused on identifying the underlying chemical reactions that can lead to self-heating and a thermal event, but specific processes are difficult to isolate because the mechanisms are complex and strongly depend on the battery materials and environmental conditions.^5,9,11^ Deng *et al.*^1^ emphasized the importance of both experimental and theoretical efforts to improve battery safety assessments, since accurate chemical simulations rely on experimentally determined reaction networks and onset temperatures to make reliable predictions.^12,13^ Researchers postulated temperature-dependent degradation reaction networks for interplay of the negative electrode with the electrolyte,^4,14^ and for the bulk electrolyte^15^ for standard mixtures using ethylene carbonate (EC), dimethyl carbonate (DMC) and lithium hexafluorophosphate (LiPF_6_). Recent studies analyzed the temperature-dependent degradation of NMC materials,^16,17^ but the mechanisms of the subsequent interfacial electrolyte oxidation during thermal abuse are less well established. In particular, the sequence and intermediates of chemical oxidation during thermal abuse, as well as the influence of open circuit potential (OCP), are still debated, motivating the present study.

At room temperature, degradation reactions at high potentials of carbonate-based electrolytes on the positive electrode (NMC or similar) are well described, and have been found to substantially contribute to solvent degradation.^18–20^ Acidic species and water are formed by electrochemical and chemical oxidation reactions, whereas the onset potential decreases with increasing temperature.^21^ Many degradation products of the oxidative decomposition of solvents, including EC, have been identified, such as water, CO, CO_2_, glycolic acid, and oxalic acid.^19,20^ The pathways and rates of these degradation reactions often depend on the potential of the positive electrode.^20,22,23^ Water and acids lead to H_2_ evolution after they diffuse to and reduce at the negative electrode.^18^ A high state of charge of the battery lowers the onset of self-heating, increases the severity of a thermal event, and leads to increased gas formation, especially H_2_.^24,25^ Reactions of electrolyte solvents at the NMC surface can be facilitated at higher voltages. So far, most focus was on low temperatures *T* < 60 °C and on combustion at high temperatures *T* > 150 °C. Yet, the intermediate region, which is crucial to understand initial self-heating, is largely unexplored.

Water contamination, whether introduced externally, *e.g.*, during production^26^ or formed by oxidation reactions,^19^ can contribute to cell degradation and potentially accelerate self-heating of a cell.^13^ Studies focusing on reaction pathways identified that water is able to alter SEI composition,^27–30^ initiate electrolyte polymerization,^18,30^ and amplify the degradation of both the conductive salt^14,31^ and the positive electrode materials.^32,33^ Furthermore, a direct correlation between water concentration and the presence of HF in the electrolyte has been observed,^14,34^ which further affects the decomposition and dissolution of SEI species at elevated temperatures. However, the low initial water concentrations in LIBs cannot fully account for the large amounts of H_2_ observed after thermal events,^25^ underlining the potential importance of reactions at the positive electrode as a source of water and acidic species for gassing and self-heating.^10^

In this study, we elucidate the oxidation pathways of EC-based electrolytes on Ni-rich NMC positive electrodes at elevated temperatures. Products and pathways of chemical oxidation during thermal abuse were determined experimentally using *in situ* high temperature-online electrochemical mass spectrometry (HT-OEMS) combined with multiple analytical post-mortem tools. We reveal a complex reaction network initiated by the interaction of EC and water on the positive electrode at cell temperatures in the range of 60 °C to 130 °C. Using density functional theory (DFT) simulations, we quantify the heat release of these reactions. These findings, in turn, connect molecular-level degradation chemistry with the self-heating behavior of a LIB, revealing the role of chemical oxidation in a thermal event.

## Experimental section

### Battery materials and formation

All experiments were performed with an EL-CELL high-temperature gas test cell similar to the PAT-Series (as presented in ref. 11). The EL-CELL equipment includes a polyether ether ketone sealing ring, a polyether ether ketone insulation sleeve, but no reference electrode was used. Electrodes were cut into disks with an 18 mm diameter using the EL-Cut. Electrode materials were provided by CustomCells Holding GmbH: the negative electrode consisted of 95% graphite, single-sided coated on copper, batch-no. A-2557; specific capacity 350 mA h g^−1^, areal capacity 2.4 mA cm^−2^. The positive electrode consists of 96% lithium–nickel–manganese–cobalt oxide (NMC811) with molar fractions of 80% nickel; 10% manganese; 10% cobalt, single-sided coated on aluminum, batch-no. K-1478, specific capacity 175 mA h g^−1^, areal capacity 2.0 mA cm^−2^. A polytetrafluoroethylene separator manufactured by Omnipore JVWP04700 with a porosity of 80% and thickness 30 µm was used.

The electrolyte was EC/DMC 50/50 (vol.-%) with 1 M LiPF_6_ from Sigma-Aldrich. Heavy water was purchased from Sigma-Aldrich with 99% D_2_O and was added with concentrations of 300 ppm and 1500 ppm. The concentration of 300 ppm was chosen based on the previous study of Lundström *et al.*^30^ on the effect of water on EC-based electrolytes and 1500 ppm to magnify water-induced effects for easier detection during analysis. Battery assembly was performed under an argon atmosphere in a glovebox with <0.1 ppm of water and oxygen.

### High temperature-online electrochemical mass spectrometry

After cell assembly, cells were connected to the HT-OEMS setup, airtightness was checked and the system was flushed with argon for 2 h before electrochemical testing. The setup consists of a Pfeiffer Vacuum GSD320 OC2 mass spectrometer, a Gamry 5000E potentiostat and a Bronkhorst EL-Flow Prestige FG-200CV10 mass flow controller. The carrier gas was argon 5.0 (<2.0 ppm H_2_O) provided by Air Liquide and applied with a flow of 0.65 mL min^−1^. The mass spectrometer was operated with a capillary temperature of 200 °C and inlet temperature of 120 °C. The energy of the electron beam was set to 70 eV. In total, 31 *m*/*z* channels were recorded with a C-SEM detector using a dwell time of 500 ms, resulting in a resolution of roughly 17.5 s per point per channel.

Two formation cycles with CC/CV charge and CC discharge at C/5 were performed at room temperature. The cut-off criterion for charging was 4.2 V, and the CV step was continued at the same voltage until current dropped below C/20; the discharging cut-off voltage was 3.0 V. A rest time of 10 min between charging and discharging was used. After the second formation cycle, the cells were charged with CC/CV to their respective cell voltage of 4.0 V, 4.2 V or 4.4 V. Current for the CC-step was C/5 and CV cut-off was C/20. For all tests, cell voltages during thermal abuse conducted are below the electrolyte's gas evolution onset as determined under room temperature conditions (4.6 V; Fig. S2).

Thermal stress was applied after at least 10 min of relaxation; heating was applied with a heat rate of 2 °C min^−1^ from room temperature (25 °C) to 132 °C. The maximum temperature of 132 °C was held for 1 h. The heat supply was turned off at the end of the described heating profile.

For data evaluation (number of measurements ≥3), the ion current signals from mass spectrometry were baseline corrected, normalized to the argon signal (*m*/*z* 36), and solvent signal subtraction was performed. For solvent signal subtraction, pure DMC was evaporated with the same temperature profile as for HT-OEMS experiments, to determine the fragmentation pattern of DMC in the mass spectrometer. A solvent signal subtraction of the signal of *m*/*z* 26, *m*/*z* 28, and *m*/*z* 44 was performed relative to *m*/*z* 90 with the fragmentation relationship from the separately measured DMC. We acknowledge that thermal degradation products of DMC can, in principle, contribute to several mass channels, in particular *m*/*z* 32 (methanol) and *m*/*z* 44 (CO_2_). These potential contributions were not subtracted during solvent background correction, which was limited to characteristic DMC fragments. The mechanistic interpretation presented here therefore focuses on relative trends and temperature-dependent changes rather than absolute signal intensities.

### Scanning electron microscopy

Electrode samples under different conditions were cut to 5 × 5 mm, fixed to sample holders using adhesive carbon tape and sputtered with a thin (<10 nm) platinum coating (EM ACE200, Leica, Germany). Scanning electron microscopy (SEM) micrographs were obtained using a Zeiss 1540 XB (Zeiss Microscopy, Germany) at an acceleration voltage of 1.3 kV. Both, InLens and SE detectors were used for imaging, to account for different surface conditions.

### X-ray diffractometry

For X-ray Diffractometry (XRD) analysis, cells were disassembled in the glovebox after the electrochemical procedure and thermal abuse, and the positive electrodes were submerged in DMC for 30 s. Kneaded clay was used to fix the samples on the inert sample holder. XRD experiments were performed within 24 h after the inert sample holder was removed from the glovebox. XRD was performed using a Bruker D8 ADVANCE with the matching airtight dome sample holder. Measurements were performed using a Cu Kα X-ray source with 1600 W (40 kV; 40 mA) in a range (2*θ*) from 10° to 90°. Sample rotation speed was set to 3 rotations per minute. A step size of 0.01° was used with a scan time of 4.5 s for each point.

### High performance liquid chromatography

For High Performance Liquid Chromatography (HPLC), one quarter of a NMC electrode was submerged in 0.5 mL of ultrapure water and stored overnight at 20 °C. The device used was a Vanquish UHPLC from Thermo Fisher Scientific. HPLC measurements were performed with an Acclaim Guard column and a HyperREZ XP carbohydrate-H + LC column, both from Thermo Fisher. The eluent used was 5 mmol L^−1^ H_2_SO_4_ in water with a flow rate of 0.6 mL min^−1^, column temperature was set to 55 °C, injection volume was 10 µL, and the UV detector wavelength was set to 210 nm. Analysis was performed with Chromeleon software. Identification and quantification were performed using external calibration standards of diluted raw chemicals.

### Density functional theory

The reaction energies and enthalpies proposed in this work were obtained using the Gaussian 16 DFT software package.^35^ Calculations were performed at both 298 K and 400 K using the B3LYP functional and cc-pVTZ basis set.^36–38^ The solvation environment was simulated implicitly using the polarizable continuum model^39,40^ with the dielectric constant set to 22.94, corresponding to values reported in the literature of a 1 : 1 mixture of EC : DMC.^41^ The NMC surface was not taken into account for the calculations.

## Results

### The role of cell voltage in thermal degradation

**Fig. 1:**
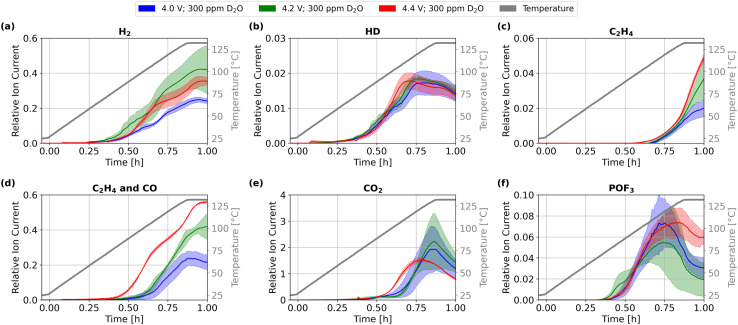
Gas evolution during thermal abuse up to 132 °C of lithium-ion cells containing 300 ppm of D_2_O dosage at OCPs of 4.0 V (blue), 4.2 V (green), and 4.4 V (red): (a) *m*/*z* 2H_2_, (b) *m*/*z* 3 HD, (c) *m*/*z* 26 C_2_H_4_, (d) *m*/*z* 28 C_2_H_4_ and CO, (e) *m*/*z* 44 CO_2_, and (f) *m*/*z* 104 POF_3_. Colored areas represent standard deviation from mean values. For additional *m*/*z* values see Fig. S3.

Previous studies emphasized increased reactivity of the positive electrode at high potentials,^22,33,42^ which may lead to earlier onset and increased severity of a thermal event.^24^ In order to identify the underlying pathway of chemical degradation for the positive electrode up to 132 °C, we conducted thermal abuse measurements on cells charged to 4.0 V, 4.2 V, and 4.4 V, corresponding to a state of charge of 77%, 100%, and 110%, respectively. The cells (graphite, NMC811, 1 M LiPF_6_ in EC/DMC) were analyzed using HT-OEMS. To differentiate hydrogen formed from the initial water content *versus* contributions from solvent oxidation, we added 300 ppm D_2_O as an isotope tracer for the initial water content. Additionally, we applied SEM, XRD, and HPLC to the samples after thermal abuse to identify solid and soluble degradation products. The gas evolution of several species during heating to 132 °C at different OCPs is shown in Fig. 1(a)–(f).

The onset temperatures for the evolution of H_2_, HD (D originating from heavy water), C_2_H_4_, and POF_3_ were independent of the OCP. Only for C_2_H_4_ at *T* > 130 °C the total evolved quantities increased with voltage. This gas is commonly attributed to SEI degradation and reformation during a thermal abuse; the behavior observed here suggests increased presence of SEI formation and degradation reactions at high OCPs and elevated temperatures. CO and CO_2_ show a further effect: an earlier onset of their evolution for 4.4 V. Additionally, during temperature increase at 4.4 V the quantities of CO increase, while CO_2_ presence decreases, indicating a general shift towards CO formation; note that C_2_H_4_ contributes to *m*/*z* 26 and *m*/*z* 28 signals, since no changes of *m*/*z* 26 signal appears before the temperature hold at 132 °C, the OCP-dependent behavior of *m*/*z* 28 signal during temperature ramping can be assigned to CO evolution. CO and CO_2_ can result from SEI decomposition. However, the absence of an increase in C_2_H_4_, *i.e.*, SEI degradation, with potential at temperatures below 130 °C suggests a different origin. The earlier evolution of CO and CO_2_ more likely originates from solvent oxidation at the positive electrode: high potentials may trigger the chemical oxidation of EC.^19,43^ Similar conclusions have been reported in previous thermal abuse studies, where the majority of CO_2_ evolution was attributed to reactions at the positive electrode.^10^

If such oxidation reactions occur, H_2_ production from the reduction of the evolving water would be expected. As only the initial water content was isotope-labeled, the hydrogen evolution corresponding to solvent oxidation should not be isotope-labeled. While the intensity of H_2_ increases for 4.2 V and 4.4 V, HD remains constant. This suggests that the additional hydrogen is formed from products of solvent oxidation, rather than the initial water content. The finding supports crosstalk-induced hydrogen evolution, in which degradation products from the positive electrode diffuse to the negative electrode.^44^ This process was observed to be amplified with increasing cell voltages,^22^ which can explain the increased evolution of both unlabeled H_2_ and CO at 4.4 V.

**Fig. 2:**
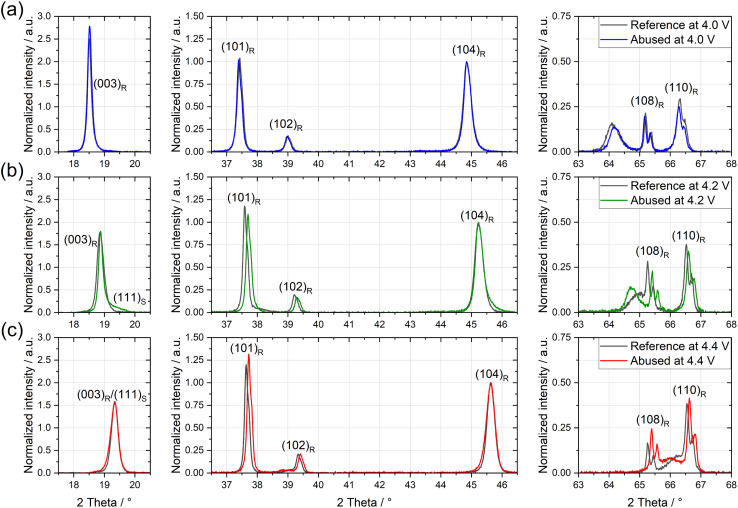
Selected regions of XRD spectra of positive electrodes before and after thermal abuse with 300 ppm D_2_O dosage at OCPs of (a) 4.0 V, (b) 4.2 V, and (c) 4.4 V. Spectra were normalized to the height of the (104) diffraction peak. Peaks were indexed to their corresponding reflection following Bak *et al.*;^8^ L = layered oxide; S = spinel indicate their corresponding structure.

To further validate that the gases originate from oxygen release from the NMC811 active material, we performed XRD analysis of the positive electrodes before and after thermal abuse to identify possible structural changes (Fig. 2).

After thermal abuse at 4.0 V, only a minor shift of peaks can be observed. As structural changes, such as phase transformation into a spinel structure or changes to the layered structure, *e.g.*, due to (de-)lithiation, would cause changes in peaks,^33^ we conclude that there are negligible structural changes after thermal abuse at 4.0 V. In contrast, electrodes subjected to thermal abuse at 4.2 V and 4.4 V show the development of shoulders on the (003) and (104) peaks (Fig. S6) and shifts of the (101), (108), and (110) peaks to higher angles (Fig. 2). By referencing our spectra to the results of Bak *et al.*,^8^ we attributed the shoulders on the (003) and (104) peaks to the appearance of (111) and (400) reflections. Additionally, a new peak of (220) reflection formed at approx. 30°. Following Bak *et al.*, these new reflections correspond to the formation of the spinel phase. The peak shifts of (101), (108), and (110) to higher angles may indicate the onset of the bulk degradation of the layered structure. They do not correspond to an increase in the lithiation of the NMC material, connected to electrochemical oxidation proposed under OCP conditions,^45^ which would correspond to shifts to lower angles in this voltage range.

*Ex situ* experiments reported that the onset temperature for bulk structural changes of NMC811 at 4.3 V *vs.* Li/Li^+^ is approximately 160 °C using XRD, whereas the onset for oxygen evolution was already detected at 135 °C.^8^ Recent studies revealed that surface changes of NMC appear at much lower temperatures than the bulk changes,^16,17^ which can explain the detection of oxygen gas before structural changes are observed in XRD.

We detected indicators of spinel phase in our full cells after abuse at 132 °C, which are significantly lower than observations of *ex situ* experiments.^8^ While XRD does not directly quantify oxygen release, the combined evidence from voltage-dependent structural reconstruction, temperature-resolved gas evolution, and prior reports on early oxygen release from NMC surfaces supports the involvement of lattice oxygen-driven oxidation processes under thermal abuse conditions. In HT-OEMS we observed no significant quantities of O_2_ gassing (see Fig. S3), but increasing quantities of CO for higher OCPs (see Fig. 1(d)). We thus suggest that released lattice oxygen likely oxidizes EC to CO, CO_2_ and other oxidation products. The onset of CO and CO_2_ evolution begins at 100 °C for both, 4.0 V and 4.2 V, and already at 80 °C for 4.4 V, see Fig. 1(d) and (e). These results suggest that the onset of structural degradation of NMC811 in full cells compared to *ex situ* experiments is reduced by 30 °C for 4.2 V, and that for 4.4 V thermal stability decreases further, highlighting a significant reduction in the thermal stability of NMC811 materials at high voltages.

For a deeper understanding of the interfacial degradation mechanism involving the formation of CO and CO_2_ from lattice oxygen release, we applied SEM imaging on the surface and analyzed surface species on the positive electrode by HPLC. In SEM, we observed a newly formed surface film on the positive electrode after thermal abuse for all OCPs; no morphological changes could be identified for different voltages on the positive electrode (Fig. S10, compare (c) to (e)–(g)). HPLC analysis revealed that the surface film contains glycolic acid and oxalic acid for 4.0 V and 4.2 V, and only glycolic acid for 4.4 V (Fig. S11). The chemical oxidation of the solvent, resulting from lattice oxygen release and formation of acidic species, has been previously reported by other studies, but has generally only been analyzed at near-ambient temperature.^18–20,22,23,44^ Only minor changes in the concentration of glycolic acid were observed for different OCPs. Oxalic acid appeared in similar but very low concentrations for 4.0 V and 4.2 V, and was absent for 4.4 V (Fig. S11). The weak dependency of acid concentration on OCP suggests that the formation of these acidic species does not correlate with the results of XRD, and thus their formation is not limited by the availability of released lattice oxygen. The mechanistic origin of our observations, including the role of pathway competition and reactant availability, is discussed in detail in the Discussion section.

Literature suggests two types of solvent oxidation pathways: the direct oxidation of EC consuming four lattice oxygen atoms to form CO_2_, CO, and water, and a stepwise oxidation of EC with a total of five lattice oxygen atoms, to first form glycolic acid, then oxalic acid, and finally CO_2_ and water.^19,43^ Based on the results provided by HT-OEMS, XRD, and HPLC, we suggest that the reaction rate of direct oxidation is determined by the potential of the NMC, whereas the reaction rate of the stepwise oxidation is limited by a different factor. Structural changes of the NMC were identified after thermal abuse at high cell voltages, where both the presence of stepwise oxidation (indicated by carboxylic acids in HPLC) and direct oxidation (indicated by increased CO_2_ and CO in HT-OEMS) were observed. In contrast, for 4.0 V, where mainly stepwise oxidation occurred, no structural changes were observed. Acid content seems to be OCP-independent and at low OCP the NMC structure is unchanged despite the acid production requiring lattice oxygen; we conclude that direct oxidation, when present, proceeds at significantly higher reaction rates than the stepwise acid production.

DFT calculations reveal that both pathways of EC oxidation are thermodynamically favorable and thus may occur when assuming triplet O_2_, as all steps have negative Gibbs energies (see Table SI 5) at room temperature, but are even more likely at 126.85 °C (400 K). This further supports the existence of both pathways. In addition, both pathways are exothermic and release significant heat, with enthalpies of −834 kJ mol^−1^ and −417 kJ mol^−1^ for direct oxidation of EC and oxidation to glycolic acid, respectively. The reaction enthalpies calculated correspond to ∼2–4 times greater heat release than SEI degradation reactions per mol EC (Δ*H*_R_ ≈ −220 kJ mol^−1^ (ref. 13)), which illustrates that solvent oxidation can act as a driving force for a thermal event.

**Fig. 3:**
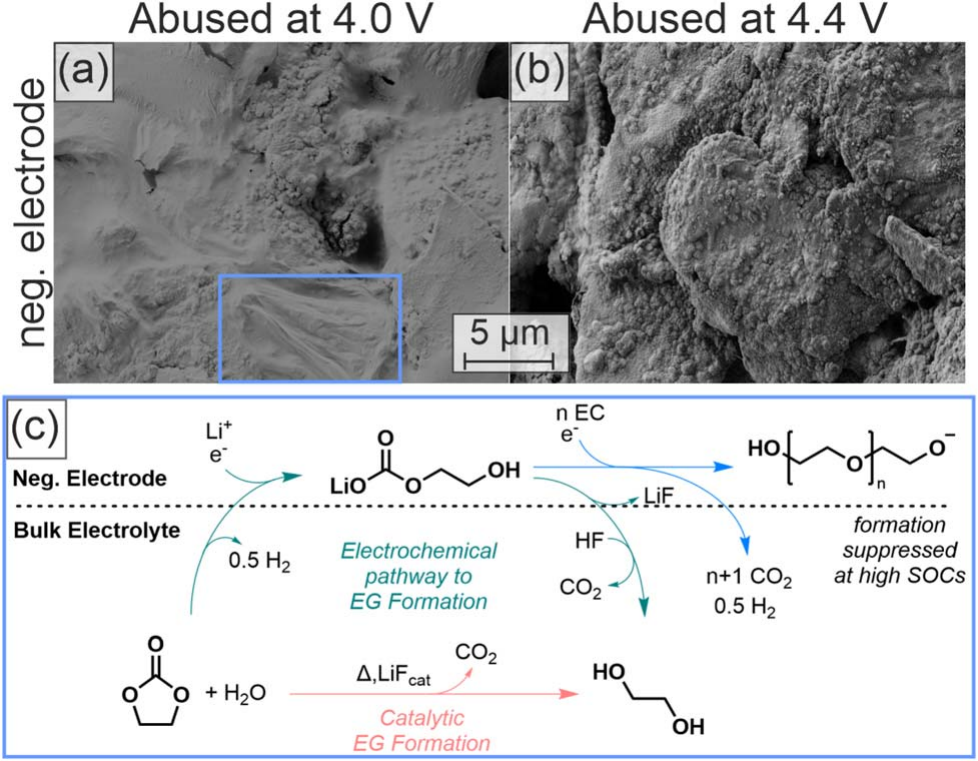
Post-mortem SEM images of the negative electrode after thermal abuse containing 300 ppm D_2_O at different OCPs and suggested mechanism for polymer formation. (a) Negative electrode after thermal abuse of cells with 300 ppm D_2_O and an OCP of 4.0 V. (b) Negative electrode after thermal abuse with 300 ppm D_2_O and an OCP of 4.4 V. (c) Reaction scheme of the polymerization processes triggered by formation (green route)^18,47^ or during thermal abuse (orange route).

Products of oxidation reactions have been reported to alter the SEI due to crosstalk reactions.^46^ To investigate such effects, we conducted SEM analysis on the negative electrodes of the cells after thermal abuse. Fig. 3(a) and (b) show the surfaces of negative electrodes after thermal abuse at 4.0 V and 4.4 V, respectively.

Cells charged to 4.0 V with 300 ppm D_2_O featured a smooth surface film that covers the graphite particles after thermal abuse. Elastic properties of this film (blue square in Fig. 3(a)), as well as bubble-like inflation of enclosed gases within the film under SEM vacuum conditions (Fig. S10(h)–(k)), can be observed. These observations suggest that the film is a polymer; previous studies identified the reduction of water and EC to form polymers and ethylene glycol (EG) (Fig. 3(c)).^18,29,48^ Additionally, polymerization initiated by salt degradation products, such as POF_3_, has been reported.^15^ However, in our HT-OEMS measurements no elevated POF_3_ formation was observed under the conditions where polymeric films were identified by SEM, indicating that salt-degradation-initiated polymerization is not supported by our experimental observations. Reaction rates for the polymer chain growth will be significantly increased at high temperatures, but the presence of water is necessary to initiate the process. The consequences of the formation of EG from water and EC will be discussed in the next chapter.

The negative electrode abused at 4.4 V with 300 ppm D_2_O (Fig. 3(b)) shows notably different surface properties with significantly increased roughness, indicating a less polymer-dominated interface. Since the same amount of heavy water was added, the absence or reduced presence of the polymerization at 4.4 V is likely due to an additional process inhibiting the initiation of polymerization or chain growth. We observed increased C_2_H_4_ and H_2_ evolution at this OCP, and thus increased and possibly competing reactions on the negative electrode. Additionally, the polymer formation proceeds *via* an anionic state^18,30^ (Fig. 3(c)), which implies that higher amounts of cations and acidic species can act as polymerization inhibitors, including carboxylic acids, phosphoric acids, and HF. Increased acid formation during thermal abuse at higher cell voltages has previously been reported;^49^ we did not observe an increase in carboxylic acid formation, but an increase in POF_3_ formation, which likely results in higher concentrations of phosphoric acids and HF.

In summary, our multi-methodical approach allowed identification of the presence of chemical oxidation of EC during thermal abuse under OCP hold. The stepwise oxidation of EC is already present at 4.0 V cell voltage, but no structural changes of the NMC811 bulk are triggered, and the quantity of the corresponding products glycolic acid and oxalic acid found after thermal abuse remains small and mainly OCP-independent. At 4.4 V, significant direct oxidation of EC was observed, which leads to significantly earlier onset of CO and CO_2_ gassing and structural changes of NMC811. Due to the strongly exothermic nature of both pathways, solvent oxidation likely plays a major role not only in a thermal event, as previously proposed, but also during the self-heating phase.

### Role of the initial water content in thermal degradation

Water impurities were previously identified to trigger degradation reactions of electrolyte components such as LiPF_6_ ^31^ and EC.^50^ To isolate the specific chemical role of the water during thermal abuse, D_2_O was added in concentrations of 0 ppm, 300 ppm, and 1500 ppm to the cells. HT-OEMS analysis was performed during formation and subsequent thermal abuse at 4.0 V. Additionally, we applied SEM, XRD, and HPLC to the samples after thermal abuse and performed DFT calculations for further analysis.

**Fig. 4:**
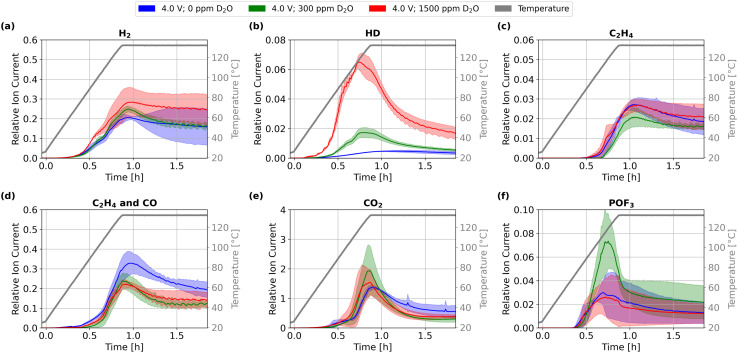
Gas evolution during thermal abuse up to 132 °C of lithium-ion cells at an OCP of 4.0 V containing 0 ppm, 300 ppm, and 1500 ppm of D_2_O dosage: (a) *m*/*z* 2 H_2_, (b) *m*/*z* 3 HD, (c) *m*/*z* 26 C_2_H_4_, (d) *m*/*z* 28 C_2_H_4_ and CO, (e) *m*/*z* 44 CO_2_, and (f) *m*/*z* 104 POF_3_. Colored areas represent standard deviation from mean values. For additional *m*/*z* values see Fig. S4.

Gas evolution during formation showed an increase only in labeled HD and D_2_ with higher D_2_O content (Fig. S1) which corresponds to the increased reduction of added water, consistent with prior findings.^29^ Water content also impacts the gas evolution during thermal abuse, as illustrated by Fig. 4(a)–(f) for selected gaseous species and different D_2_O concentrations.

During thermal abuse, higher concentrations of D_2_O cause a significant increase in the total amount of HD (*m*/*z* 3); the amount of D_2_ is 10-fold less intense than that of HD, but shows a similar dependence (Fig. S4). H_2_ shows no increase during the heat-up phase for higher D_2_O dosages, suggesting that the evolution is not affected by D_2_O dosing; minor fragmentations of HD and D_2_ contribute to the signal of *m*/*z* 2, but the main signal should originate from H_2_. H_2_ formation stems from the reduction of water, alcohols or acids from sources other than the added water content of the electrolyte, since water has been isotope-labeled. The observation that the presence of H_2_ is largely unaffected by the D_2_O dosage, which is a significant portion of the initial water content at 1500 ppm, reveals that other sources for H_2_ evolution significantly contribute to the formed amount.

For the cases of 300 ppm and 1500 ppm, the evolution of H_2_ exhibits similar temperature-dependent behavior to HD until approximately 120 °C, both showing increasing signal intensities. Beyond this point, a clear separation of the curves occurs: H_2_ evolution continues to rise until the maximum temperature and remains elevated during the temperature hold. In contrast, HD exhibits a maximum intensity at 120 °C and then decreases throughout the rest of the experiment. These findings suggest that during thermal abuse, initially, the residual water impurities, including the added D_2_O, are reduced. Once consumed, here above 120 °C, a different H_2_-producing pathway is more prevalent. Based on our results in the previous section, we propose that the ongoing release of H_2_ above 120 °C primarily results from the ongoing crosstalk-based reduction of water and acidic species at the negative electrode, both formed by solvent oxidation at the positive electrode. During oxidation, the hydrogen from C–H bonds of EC is released as water, which corresponds to unlabeled hydrogen. This explains the increased presence of H_2_ for cells with higher OCPs and initial water contents above 120 °C, likely marking the onset temperature of the stepwise oxidation.

C_2_H_4_ evolution is unaffected by the presence of water, suggesting that EC reduction associated with SEI degradation and reformation is not significantly affected by the water content. A slight increase in CO and CO_2_ production during the temperature hold at 132 °C was observed in the absence of D_2_O dosage, indicating increased direct solvent oxidation. This suggests an increased occurrence of this process independent of the presence of water. A pathway explaining this observation will be discussed in a later section. POF_3_ evolution increased for cells with 300 ppm of D_2_O compared to cells with 0 ppm and 1500 ppm, whereas 0 ppm and 1500 ppm showed similar behavior and quantities. This deviating trend suggests that the interaction of LiPF_6_ with water cannot be fully described by hydrolysis alone in the case of full cells, as an increase with increasing water content would otherwise be expected.^51^ These findings point toward more complex interactions, as also suggested by previous studies,^52^ and need further investigation in future studies.

**Fig. 5:**
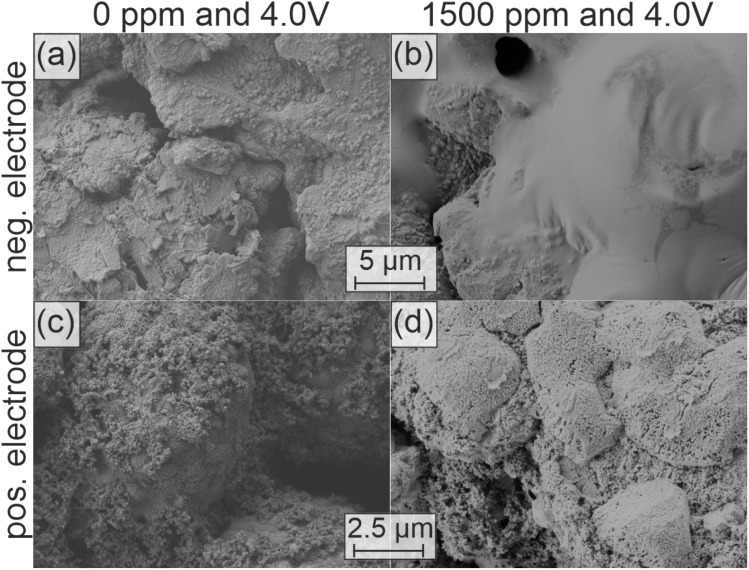
Post-mortem SEM images of the electrode surfaces after thermal abuse at an OCP of 4.0 V with different D_2_O contents: (a) negative and (c) positive electrodes after thermal abuse of cells with 0 ppm D_2_O. (b) Negative and (d) positive electrodes after thermal abuse of cells with 1500 ppm D_2_O.

To further validate the source of the additional H_2_ production, we conducted post-mortem SEM analysis on both the negative and positive electrodes after thermal abuse. Fig. 5 shows the surface morphology of the negative and positive electrodes with 0 ppm in (a, c) and 1500 ppm D_2_O (b, d) after thermal abuse.

After thermal abuse the negative electrode with 1500 ppm D_2_O shows a polymer, similar to the test with 300 ppm (see Fig. 3(a)), whereas polymers are not visible for 0 ppm. This further confirms that the mechanism of polymerization is dependent on the initial water content, as also suggested in the reaction scheme in Fig. 3(c).

On the positive electrode, a significantly more pronounced surface film is visible for 1500 ppm D_2_O dosage compared to 0 ppm. XRD analysis of positive electrodes did not reveal any structural changes for all D_2_O concentrations (Fig. S8 & S9). However, dissolution of the film and HPLC analysis revealed a strongly increased presence of glycolic acid with increasing D_2_O dosage, while the oxalic acid concentration remained unchanged (Fig. S5). This indicates that the proposed underlying chemical oxidation of EC is enhanced by water, though unlikely through direct participation, since water is also an oxidation product. Instead, we suggest an indirect water-facilitated pathway, in which EC and water react to form EG and CO_2_. This chemical reaction has been shown to appear at temperatures of 120 °C and is catalyzed by the presence of inorganic salts.^50^

Two pathways linking EG production to the presence of water were suggested in the past^30,50^ (Fig. 3c). To validate that EC may directly react to EG, we investigated the pathway proposed by Hu *et al.*, which suggests a salt-based catalytic ring-opening of EC with water to form EG.^50^ An EC/H_2_O/LiF mixture was heated to 80 °C for 2 h. We observed significant formation of CO_2_ and EG (Section S2.5), confirming that both products are formed from EC in the presence of water under the battery abuse conditions in HT-OEMS. LiF, which catalyses this reaction (see the mechanism in Fig. S12) has a low solubility in the battery electrolyte.^50,53^ We observed the formation of the salt decomposition product POF_3_ during HT-OEMS measurements for all cells. The reaction is accompanied by HF formation.^54^ HF can overtake the catalytic role of LiF under the battery conditions, promoting the suggested EG formation.

At room temperature, the oxidation of EG was previously shown to start at cell voltages as low as 3.5 V, whereas EC oxidation starts only at higher voltages.^55^ To further validate the role of chemical oxidation of EG, we analyzed products formed by pure EG as well as the electrolyte at 125 °C in the presence of delithiated NMC (corresponding to 4.0 V cell voltage), in the absence of the negative electrode. Similar isolated-electrode approaches have been used in previous studies to identify temperature-driven oxidation products originating from delithiated cathode materials.^10,56^ HPLC analysis revealed the formation of glycolic acid, oxalic acid, and acetic acid after thermal abuse of each sample. Pure EG electrolyte shows 15-fold more oxalic acid than the electrolyte, 5-fold more acetic acid, and the same amount of glycolic acid (see Table S3). Acetic acid was only found in these experiments, but not in full cell investigations. These results support the suggested pathways of EG decomposition leading to carboxylic acid formation at delithiated NMC, where glycolic acid can be further oxidized to oxalic acid. Additionally, it was confirmed that EG is more reactive towards oxidation than EC.

In summary, our results revealed how the initial water content of a LIB lowers the onset potential and accelerates high-temperature chemical oxidation. EC can undergo a ring-opening reaction with water, resulting in the formation of EG. EG oxidation leads to water formation, creating an autocatalytic cycle. Additionally, EG is less stable than EC and oxidizes faster on NMC at high temperatures, leading to accelerated heat evolution and gassing. This directly links control of water levels during cell production to potential battery safety hazards.

## Discussion

**Fig. 6:**
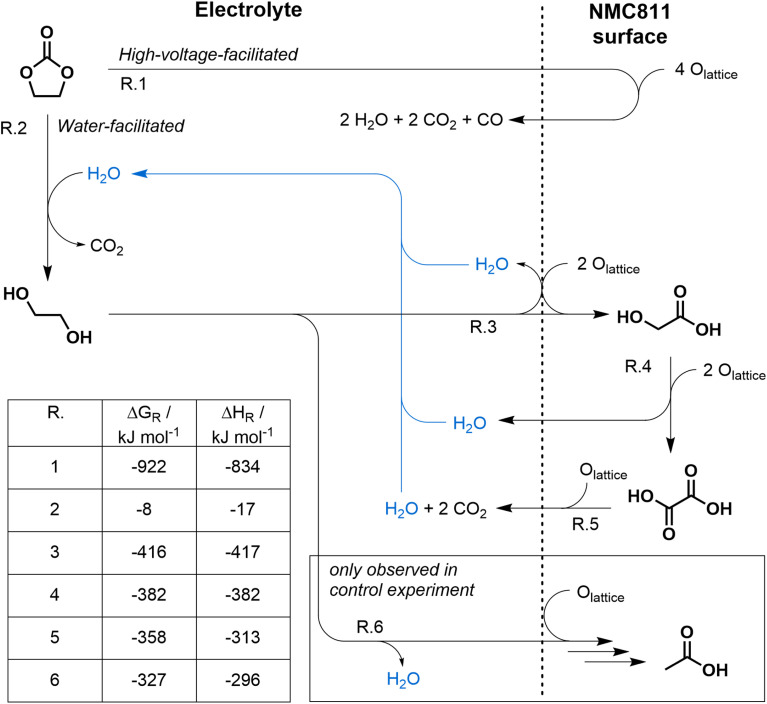
Reaction scheme for thermally induced high voltage facilitated (direct) and water facilitated (stepwise) chemical oxidation of EC at Ni-rich NMC electrodes based on the degradation products found in this study (see Fig. 1, 4, and S11). The pathway involving water is marked in blue. The table lists Gibbs free energies and reaction enthalpies at 400 K and assuming triplet oxygen for oxidation reactions.

Throughout this study, two distinct pathways for thermally induced chemical oxidation of EC were observed, with direct oxidation triggered by high cell voltages and partial oxidation by water impurities. While contributions from linear carbonates such as DMC cannot be fully excluded under thermal abuse, the observed gas and liquid-phase products indicate that EC-derived oxidation pathways dominate under the investigated conditions. The corresponding postulated reaction network is displayed in Fig. 6 and discussed in the following. A more detailed version including intermediates is shown in Fig. S12.

After thermal abuse at 4.2 V and 4.4 V, structural changes of NMC811 were observed in XRD (see Fig. 2), likely coupled to the release of lattice oxygen. The elevated voltage and oxygen availability promote direct EC oxidation, leading to a lower onset temperature of 80 °C for CO and CO_2_ evolution at 4.4 V, as observed in HT-OEMS (see Fig. 1(d)). Direct oxidation consumes four lattice oxygen atoms in a single step, and hence needs high oxygen availability (R.1). Notably, the literature reports high activation energies for the direct EC oxidation,^57^ supporting that it is only feasible at high OCPs and/or temperatures, where sufficient reactive oxygen is available. Compared with *ex situ* measurements, our full cell results show a 30 °C reduction in the onset temperatures for NMC structural degradation and lattice oxygen release.^8^ These findings underline the importance of *in situ* approaches, as electrode degradation can be significantly influenced by the presence of the electrolyte.

Already at lower voltages and lower temperatures than needed for direct oxidation, step-wise EC oxidation commences: EC undergoes ring-opening reactions at temperatures as low as 80 °C with water, which leads to the formation of EG^50^ (R.2). Once EG is formed, it is likely oxidized, producing water (R.3, R.4 & R.5), which is subsequently available for further EC ring-opening reactions producing more EG (R.2). EG formation and oxidation, consuming and producing water, can thus be seen as a water-catalysed degradation cycle of EC. Water appears to be the rate-limiting component, as evidenced by the increased quantities of carboxylic acids with increased water whereas higher voltages did not have an impact. Higher water presence increases the reaction rates for EG and glycolic acid formation, highlighting the critical importance of maintaining low moisture levels during production.

At 4.4 V and 300 ppm D_2_O, both stepwise and direct oxidation were observed concurrently, resulting in structural changes of NMC811. At 4.0 V, we observed similar concentrations of carboxylic acids, but a significantly lower amount of direct oxidation products compared to 4.4 V, and no apparent structural changes in the NMC811 electrode. These findings indicate that the structural changes of NMC811 at 4.4 V are due to excessive consumption of lattice oxygen *via* the direct oxidation pathway. A larger presence of direct oxidation should correspond to a higher yield of water and, in turn, an increase of the stepwise oxidation – yet this was not observed. We attribute this to the relative concentrations of the reactants water, EG, and EC. The low water concentration likely limits the LiF-catalyzed conversion of EC to EG and CO_2_. Oxidation of EG is able to occur at low concentrations at 4.0 V due to a lack of competing reaction partners for lattice oxygen. When the voltage is increased to 4.4 V, which is above the oxidation potential of EC, the high concentration of EC allows for the direct oxidation pathway to dominate.

While the crystallographic phase transformation of NMC is well understood, the chemical state of the associated oxygen release from the lattice is still under discussion. Theoretical studies on solvent oxidation revealed high theoretical activation energies for the reactions.^57^ In order to explain the experimentally observed appearance of oxidation reactions, oxygen release from NMC in the singlet state has been suggested.^58^ However, more recent studies have revealed that singlet oxygen alone is insufficient to cause EC oxidation.^59^ The Gibbs free energies and reaction enthalpies for all reactions in Fig. 6 were calculated using DFT assuming triplet oxygen. Since singlet oxygen is in an energetically excited state compared to triplet oxygen, Gibbs free reaction energies including those of singlet oxygen will always be more negative and thus more favorable. All three steps involved in the step-wise oxidation of EG are also thermodynamically favorable for triplet oxygen, and show significant heat release (Δ*H*_R_ = −417 kJ mol^−1^ to −313 kJ mol^−1^ per step). They are thus likely to occur, and can promote self-heating when taking place at high rates.

The HT-OEMS results revealed that, at cell voltages of 4.4 V, direct EC oxidation exhibits earlier onset and a higher reaction rate. This reaction shows significantly higher heat release (Δ*H*_R_ = −834 kJ mol^−1^) than stepwise oxidation, underlining the significant safety hazard of higher cut-off voltages for cells with EC-based electrolytes. Recent studies presented enhanced stability against the thermal event when EC is removed from the electrolyte in graphite/NMC811 cells;^60,61^ this may be directly linked to the absence of exothermic oxidation reactions driven by EC and its decomposition product EG.

## Conclusion

In this work, we applied a multi-method approach combining HT-OEMS, SEM, XRD, HPLC, and DFT to unravel the chemical oxidation pathways of EC-based electrolytes during thermal abuse of LIBs. Full cells with varied OCPs and D_2_O content were analyzed to identify degradation pathways leading to gas evolution, soluble products, surface film formation, and structural changes of the positive electrode's active material. This allowed us to establish a detailed reaction network and to confirm the thermodynamic feasibility of the underlying processes.

Our analysis revealed two distinct mechanisms of thermally induced oxidation: (i) a water-facilitated ring-opening reaction of EC yielding EG, which can be oxidized to form multiple carboxylic acids in the presence of water already at moderate voltages of 4.0 V, and (ii) a direct EC oxidation to CO, CO_2_, and water at higher voltages from 4.2 V. The reaction rate of pathway (i) is limited by water concentration, and thus the production process and water-producing or consuming aging reactions. Pathway (ii) revealed that the onset of CO/CO_2_ evolution from oxidation in full cells is lowered from 100 °C to 80 °C, as the cell OCP increased from 4.2 V to 4.4 V. The results strengthen recent findings^16,17^ that interfacial reactions of the positive electrode appear at much lower temperatures than bulk degradation and identify the subsequent degradation pathways of the electrolyte molecules.

DFT calculations revealed that the suggested pathways are thermodynamically favorable and highly exothermic, with reaction enthalpies of −416 kJ mol^−1^ for the first oxidation step of the water-driven process and −834 kJ mol^−1^ for direct EC oxidation. Such high negative values highlight the potential for chemical oxidation reactions to drive heat release and gas evolution already at temperatures as low as 80 °C to 100 °C during thermal abuse. Our findings emphasize the complex interplay between electrolyte composition, surface chemistry and operating conditions for the self-heating phase of lithium-ion batteries. By establishing a detailed reaction network that connects lattice oxygen release, solvent oxidation, and interfacial degradation, this work reveals how EC and water govern the onset of self-heating and a thermal event. The findings support recent strategies advocating the removal or replacement of EC to reduce battery safety hazards.^60,61^ The reaction network presented herein provides crucial information for thermo-chemical modeling, which can incorporate our findings to describe OCP-dependent thermal runaway behavior in future studies.^12,13^

Beyond mechanistic insight, our findings demonstrated that there might be an increased safety risk at higher cut-off voltages for batteries containing EC electrolytes. By bridging atomistic thermodynamics with *operando* analysis and cell-level outcomes, this work provides a framework for OCP-resolved safety models and knowledge-driven electrolyte design.

## Author contributions

Leon Schmidt: conceptualization, data curation, formal analysis, investigation, methodology, writing – original draft. Kie Hankins: conceptualization, formal analysis, supervision, writing – review & editing. Jorge Valenzuela: data curation, formal analysis, methodology. Rene Windiks: conceptualization, writing – review & editing. Adrian Lindner: conceptualization, formal analysis, investigation. Ruth Witzel: conceptualization, formal analysis, investigation. Yuchen Qiu: formal analysis, investigation. Edwin Knobbe: writing – review & editing, project administration. Ulrike Krewer: conceptualization, supervision, project administration, writing – review & editing.

## Conflicts of interest

There are no conflicts to declare.

## Supplementary Material

SC-017-D6SC00426A-s001

SC-017-D6SC00426A-s002

## Data Availability

The data underlying this study are openly available in the KITopen repository at https://doi.org/10.35097/dwd5j2arf73vkkq8. Supplementary information (SI): additional information on OEMS during formation and thermal abuse, XRD, SEM, HPLC, and DFT analysis, as well as detailed reaction steps for the suggested oxidation reactions. See DOI: https://doi.org/10.1039/d6sc00426a.
