# Supplementary material for: High-temperature chemical oxidation pathways in lithium-ion batteries: mechanistic insights into ethylene carbonate decomposition
Source: Chem Sci. 2026 Mar 9;17(18):9049–60. doi: 10.1039/d6sc00426a (PMC12990432; doi:10.1039/d6sc00426a)
Supplement: SC-017-D6SC00426A-s001 [file SC-017-D6SC00426A-s001.pdf]

Supplementary Information (SI) for Chemical Science.
This journal is © The Royal Society of Chemistry 2026

High-Temperature Chemical Oxidation Pathways in Lithium-ion Batteries: Mechanistic insights into Ethylene Carbonate Decomposition

Leon Schmidt[a], Kie Hankins[a], Jorge Valenzuela[a], Rene Windiks[b], Adrian Lindner[a], Ruth Witzel[a], Yuchen Qiu[a], Edwin Knobbe[c], Ulrike Krewer[a,*]

[a]*Institute for Applied Materials – Electrochemical Technologies, Karlsruhe Institute of Technology, 76131, Karlsruhe, Germany*
[b]*Materials Design SARL, 42 Avenue Verdier, 92120 Montrouge, France*
[c]*BMW Group, Battery Cell Competence Center, 80788 Munich, Germany*

Abstract

A thermal event remains a safety challenge for lithium-ion batteries due to the self-reinforcing nature of the exothermic reactions occurring at elevated temperatures. Higher states of charge have been shown to exacerbate the onset and severity of a thermal event. For cells containing Ni-rich layered oxide-based electrodes, this has been attributed to the increased instability of the material leading to lattice oxygen release. The degradation reactions on the electrode/electrolyte interface triggered by this oxygen remain insufficiently understood. In this study, we investigate high-temperature degradation pathways of ethylene carbonate (EC)-based electrolytes in contact with Ni-rich positive electrode active materials up to 130 °C. By combining in-situ high-temperature online electrochemical mass spectrometry with post-mortem analyses, we identify and validate key degradation intermediates and products. Two distinct EC oxidation pathways are revealed: one activated at high voltages, and the other one initiated by traces of water impurities. Complementary density functional theory calculations show the reactions are thermodynamically favorable and quantify the heat release associated with each pathway. Both pathways produce significant heat and lead to gassing of CO_2 and H_2. These findings suggest significant contribution of EC to thermal gas evolution and exothermicity under abuse conditions, thereby establishing a mechanistic link between electrolyte chemistry and thermal events. This integrated experimental-computational approach provides critical insights to guide improved electrolyte formulations and predictive thermal models.

Keywords: battery safety | online electrochemical mass spectrometry | chemical oxidation | thermal event | acid formation

*Corresponding author: ✉ ulrike.krewer@kit.edu

Supplementary Information

1. Online Electrochemical Mass Spectrometry

1.1. Electrochemical data of formation

All cells were processed with two cycles of formation of C/5 in online electrochemical mass spectrometry (OEMS). Tab. S1 contains the coulombic and energy efficiencies of the first and second discharge/charge of the first and second cycle of all tested cells.

Tab. S1: Average values and standard deviations (number of cells≥3) of formation efficiencies with different heavy water concentrations in the electrolyte.

Water concentration	Coulombic Efficiency / %	Energy Efficiency / %
0 ppm and 1st cycle	83.6 ± 0.2	82.4 ± 0.4
300 ppm and 1st cycle	83.9 ± 0.4	82.8 ± 0.6
1500 ppm and 1st cycle	84.1 ± 0.2	82.7 ± 0.3
0 ppm and 2nd cycle	99.6 ± 0.2	97.6 ± 0.3
300 ppm and 2nd cycle	99.6 ± 0.3	97.5 ± 0.3
1500 ppm and 2nd cycle	99.6 ± 0.1	97.5 ± 0.1

Slight increases in efficiencies were observed during the first cycle when higher water amounts were present. In the second cycle, no differences in efficiencies can be observed.

1.2. Online electrochemical mass spectrometry during formation

During formation, gas analysis with OEMS was performed for all cells. Fig. S1 shows the gas evolution during the first cycle for cells with 0 ppm, 300 ppm and 1500 ppm D_2O in the electrolyte. We attributed the m/z values to the following species: m/z 2 H_2; m/z 3 HD; m/z 4 D_2; m/z 16 CH_4; m/z 26 C_2H_4; m/z 28 C_2H_4, and CO; m/z 44 CO_2; m/z 104 POF_3.

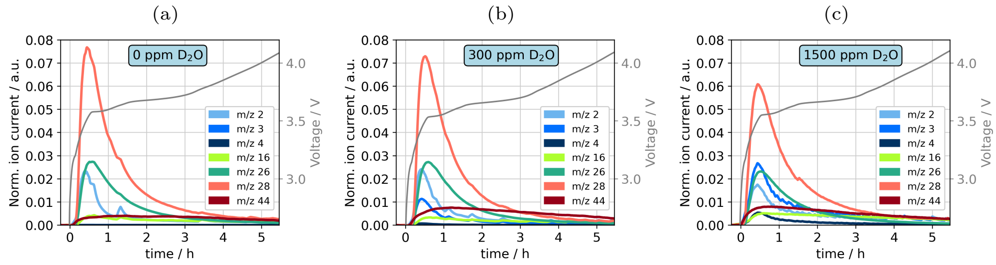

Fig. S1: Gas evolution during the first charging with different D_2O concentrations of (a) 0 ppm, (b) 300 ppm, and (c) 1500 ppm.

All cells show gas evolution of H_2, CH_4, C_2H_4, CO, and CO_2. The determination of the amount of evolving CO is not possible due to the unknown fragmentation pattern of C_2H_4 for this study. Additionally, quantification is not possible due to the application of isotope-labeling, which can shift species containing hydrogen atoms to other m/z values. When D_2O is added, we observed that for 1500 ppm m/z 3 exceeds the intensity of m/z 2,

revealing that the added D_2O significantly influences the formation process.
During the open circuit potential (OCP) variations, we increased the cell voltage up to 4.4 V. To identify if this cut-off voltage already leads to gas evolution from electrolyte oxidation, we performed overcharge experiments to 5.0 V after the formation with D_2O concentration in the electrolyte. For the overcharge experiment (Fig. S2), the same two formation steps were performed for cells with 300 ppm D_2O. Subsequently, overcharge was performed with 0.1C to 5.0 V. The results are shown in Fig. S2.

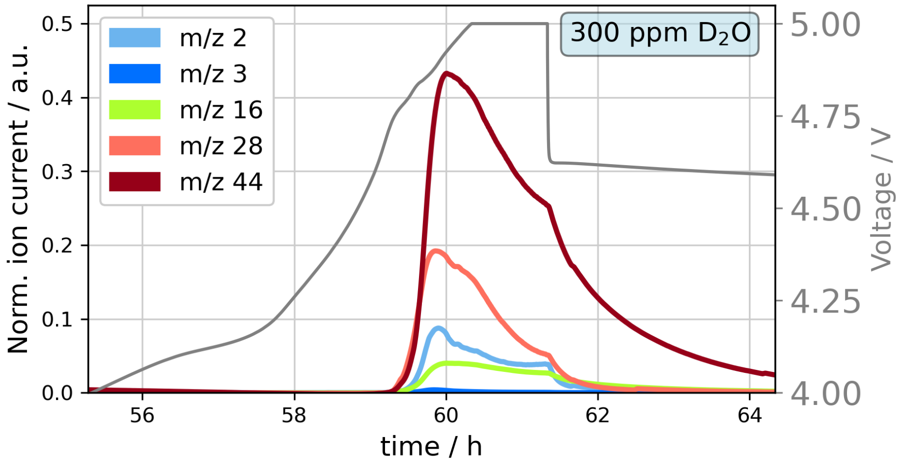

Fig. S2: Gas evolution during charging to 5.0 V at room temperature after formation with 300 ppm D_2O dosage to the electrolyte.

We detected the gases H_2 (m/z 2), CH_4 (m/z 16), CO (m/z 28) and CO_2 (m/z 44). No C_2H_4 (m/z 26; m/z 28) was detected since no increase of m/z 26 was shown. Initial gassing was observed at a voltage of approximately 4.7 V. This shows that a cell voltage of 4.4 V and below does not lead to gassing for our materials at room temperature.

1.3. Role of the cell voltage in thermal degradation - high temperature-online electrochemical mass spectrometry

Fig. S3 shows an overview of the selected m/z value during thermal abuse for increasing cell voltages of 4.0 V, 4.2 V, and 4.4 V with 300 ppm D_2O. For signals at m/z 2,

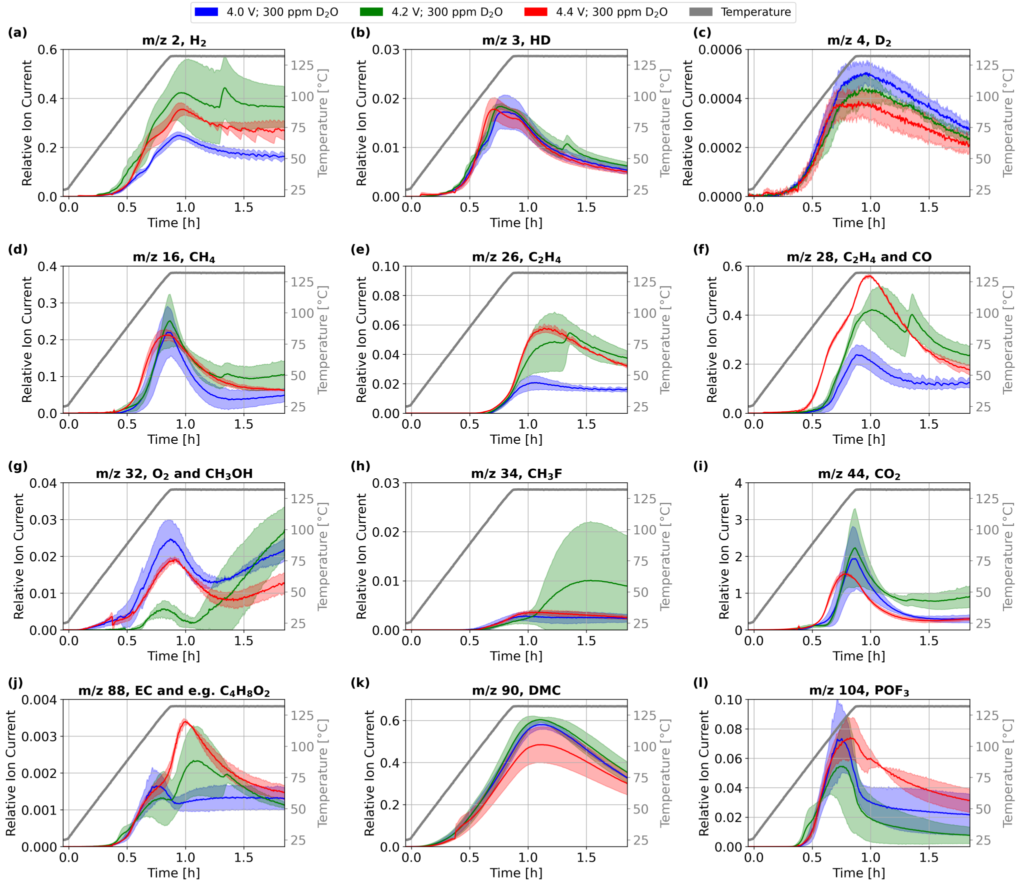

Fig. S3: Overview of selected gases using their corresponding m/z values during thermal abuse of cells with 300 ppm D_2O and OCPs of 4.0 V, 4.2 V, and 4.4 V.

an increase can be observed for 4.2 V and 4.4 V compared to 4.0 V, while m/z 3 and m/z 4 show similar signals for all OCPs. This observation was already discussed in the main text and the behavior was attributed to crosstalk reactions: at higher voltages the formation of water and acid species on the positive electrode is amplified. The formed species diffuse to the negative electrode, where they are reduced under H_2 evolution. The hydrogen is not isotope-labeled, since the protons stem from the solvent molecules, see also Fig. S13.

For CH_4 (m/z 16), only minor changes can be observed. CH_4 originated from dimethyl carbonate (DMC) reduction reactions, e.g., for solid electrolyte interphase (SEI) refor-

mation. In the case of C_2H_4 (m/z 26) an increase for 4.2 V and 4.4 V can be observed compared to 4.0 V. The formation of C_2H_4 is connected to SEI decomposition and reformation reactions caused by the decomposition and dissolution of the primary SEI [1]. For higher OCPs, higher rates of oxidation were observed (see main text), leading to the formation of water, which can damage or dissolve SEI species. Subsequent ethylene carbonate (EC) reduction leads to the additional evolution of C_2H_4. DMC reduction (m/z 16) does not change significantly, suggesting that reformation by EC reduction is dominant in this process.
m/z 28 also increases for higher OCPs, these changes can partially be assigned to the increase of C_2H_4, discussed for m/z 26. However, increased values can also be observed during the early ramping (especially for 4.4 V series), where no changes are observed for m/z 26. We attribute these changes to CO evolution, which we concluded in the main text to originate from EC oxidation.
In the case of CO_2 (m/z 44), an earlier onset and reduced quantities can be for 4.4 V; the earlier onset originated from the oxidation processes, where EC oxidation leads to CO and CO_2 evolution. The reduced CO_2 quantities can be explained, when combining with the SEM analysis from the main text (or Fig. S10 (j) to (k)), where a polymer on the surface of the negative electrode for 4.0 V, and 4.2 V was observed.

$$(CH_2OH)_2 + nEC \longrightarrow H(OC_2H_4)_{n+1}OH + nCO_2 \uparrow \quad (1)$$

For the cells showing polymer formation, it is likely to be a major source for CO_2 evolution. Since the polymer is absent for 4.4 V, reduced CO_2 quantities are expected.
POF_3 (m/z 104) only shows minor changes during ramping, but the evolution of the species during the holding time is increased for 4.4 V. Previous studies suggested that the decomposition of the conductive salt $LiPF_6$ with water resulting in POF_3, after Eq. 2 and 3, is very slow even at elevated temperatures [2, 3].

$$LiPF_6 \rightleftharpoons LiF + PF_5 \quad (2)$$

$$PF_5 + H_2O \longrightarrow POF_3 + 2HF \quad (3)$$

$$(4)$$

At high potentials, e.g. the series with 4.4 V in this study, a contribution to the formation POF_3 due to the formation of protons resulting from electrochemical oxidation (even under OCV conditions) was suggested [4]. Based on the increase in POF_3, shown here for very harsh conditions (cell temperature 132 °C and cell voltage 4.4 V) compared to milder conditions (cell temperature 132 °C and cell voltage 4.0 V), this process appears to only have a minor contribution.
The evolution of CH_3F (m/z 34) can be attributed to a subsequent reaction of POF_3 with DMC to form POF_2OCH_3 and shows a similar during ramping behavior for all OCPs. During temperature hold, a major increase is observed for one of the measurements of 4.2 V; such an increase is only observed for one cell, we could not identify any specific reason.
Oxygen and methanol evolution (m/z 32) cannot be separated during HT-OEMS. However, the behavior during the experimental time of m/z 32 and m/z 34 is very similar. Methanol can react with POF_3 as well to form POF_2OCH_3. This suggests that methanol evolution is more likely. For the solvent evaporation, we observed similar behavior for all

experiments with DMC (m/z 90); slight changes may originate from pressure differences due to other gases evolving in the cell. For EC (m/z 88), we observed different behavior during the holding phase, an unexpected result since evaporation is usually very reproducible. We believe that another species is observed at higher temperatures. Zhang et al. observed the appearance of 1,4-Dioxane ($C_4H_8O_2$) during thermal abuse with similar EC-based electrolyte [5] with lithium iron phosphate electrodes, but not for NMC. The potential observation of this molecule suggests that in full cell experiments 1,4-Dioxane may appear due to crosstalk interaction and that with increasing OCP more complex molecules may evolve.

1.4. *Role of the initial water content in thermal degradation - high temperature-online electrochemical mass spectrometry*

Fig. S4 shows an overview of the selected m/z value during thermal abuse for increasing D_2O contents 0 ppm, 300 ppm, and 1500 ppm at an OCP of 4.0 V. As described in

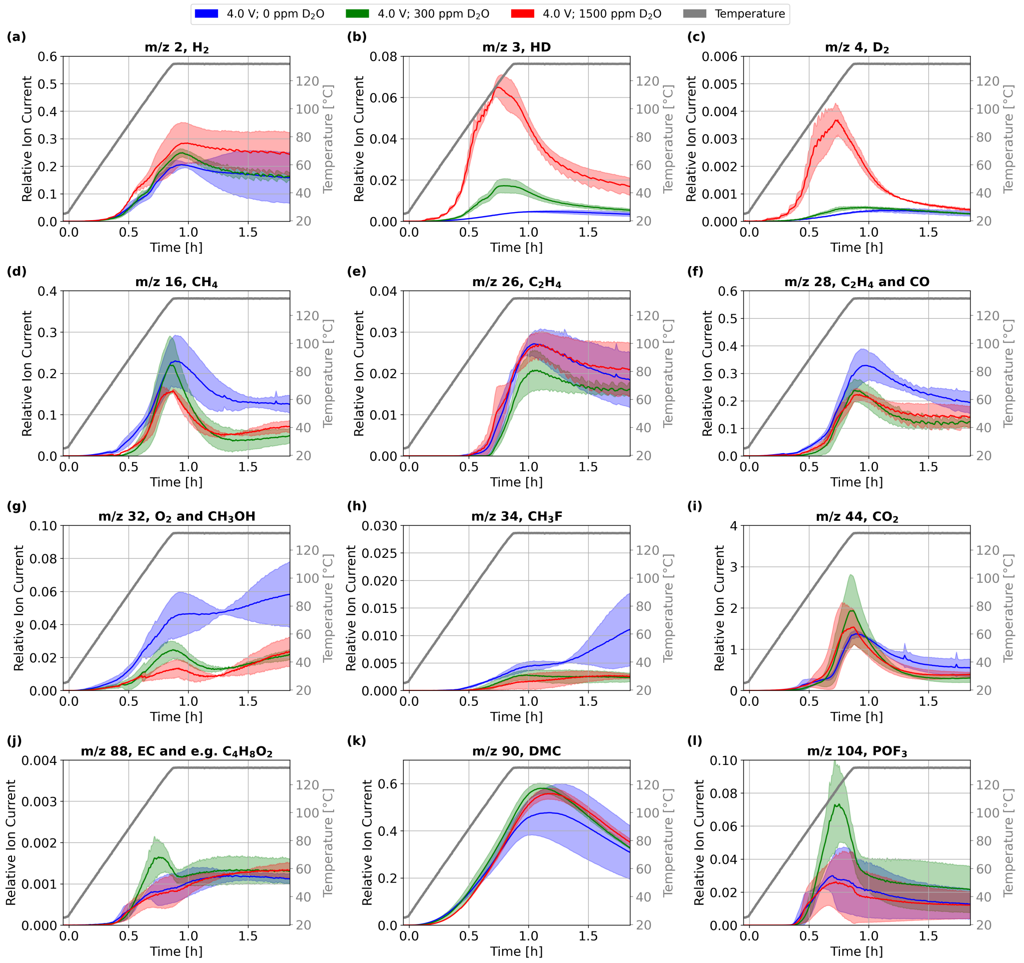

Fig. S4: Overview of selected gases using their corresponding m/z values during thermal abuse of cells at an OCP of 4.0 V and with 0 ppm, 300 ppm, and 1500 ppm D_2O dosage.

the main text, D_2 shows a significant increase due to increasing dosage of D_2O, which is expected.

CH_4 and CH_3F show increased signals for 0 ppm D_2O, which can be an indicator for increased reactivity of DMC in the absence of water. CH_4 is formed during DMC reduction to reform SEI [1]. In the presence of water, the reduction of water to LiOH or Li_2O could be dominant. In the case of CH_3F, we see an increase of the component for cells without water dosage. Previous studies described that reactions of POF_3 with DMC and water compete with each other [6]. This can explain the increased presence of CH_3F in

the absence of water.
For O_2 and methanol (m/z 32), a similar behavior to CH_3F can be observed.
For EC (m/z 88) and DMC (m/z 90), similar evaporation behaviors can be observed.

2. Post-mortem analysis

2.1. Role of the cell voltage in thermal degradation - X-ray Diffraction

Fig. S5 shows XRD spectra from 2θ of 10° to 90° for samples at different OCPs before and after thermal degradation.

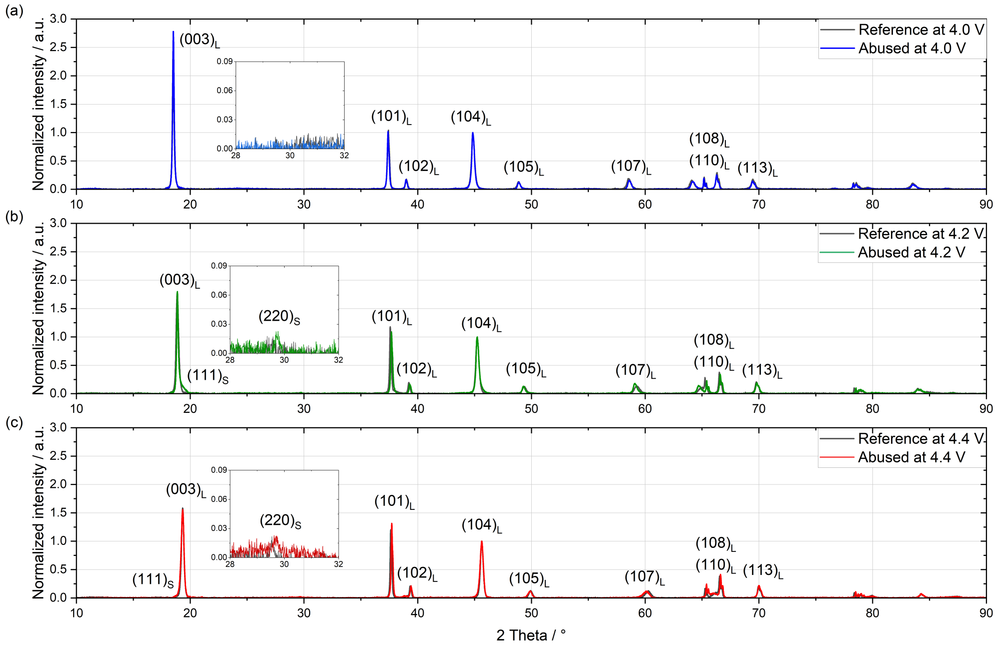

Fig. S5: XRD spectra from 2θ of 10° to 90° of NMC811 of cells with 300 ppm D_2O dosage and at OCPs (a) 4.0 V, (b) 4.2 V, and (c) 4.4 V before and after thermal abuse. Peaks were indexed to their corresponding reflection following Bak et al. [7]; L = layered oxide; S = spinel indicate their corresponding structure.

Fig. S5 shows XRD spectra from in the region of (003) and (111) reflections for samples at different OCPs before and after thermal degradation. After thermal abuse, a shoulder developed on the peak corresponding to (003) reflection of layered oxides before thermal abuse [7] for cells with OCPs of 4.2 V and 4.4 V (Fig. S6 (a)). The shoulder likely originates from a second peak emerging from the (111) reflection of spinels at approximately 2θ=19° [7]. For 4.4 V, the center of the peak of the (003) reflection is at approximately 2θ=19.4°. The peak of the (111) reflection overlaps with the peak of (003) reflection, producing the observed peak asymmetry after thermal abuse (Fig. S6 (b)).

Additionally, Fig. S7 shows a magnification of the reflections (105), (107) and (113) for the OCP variations.

Only minor shifts are observed for these peaks due to thermal abuse at all OCPs for the (105) and (113). (107) shows shifts towards lower angles for 4.2 V and 4.4 V. The significant shift of (101) reflection and the formation of e.g. (220) reflection are discussed in the main text.

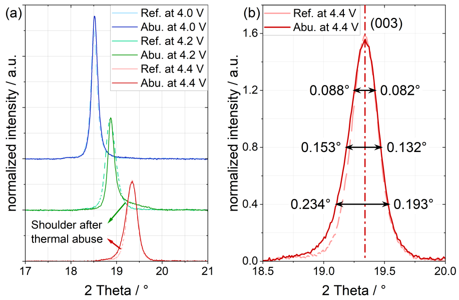

Fig. S6: XRD spectra in the region of (003) and (111) reflections: (a) NMC811 of cells with 300 ppm D_2O dosage and at OCPs of 4.0 V, 4.2 V, and 4.4 V before and after thermal abuse and (b) NMC811 of the cell with 300 ppm D_2O dosage and at an OCP of 4.4 V with information of peak symmetry

2.2. Role of the initial water content in thermal degradation - X-ray Diffraction

Fig. S8 shows XRD spectra from 2θ of 10° to 90° for samples with different water contents after thermal degradation.

Additionally, Fig. S9 shows a magnification of the reflections (003), (101), (102), (104) and (108), (110) for the OCP variations.

Only minor shifts are observed for all peaks due to thermal abuse at all water contents. This suggests that there are no structural changes to the bulk NMC811 after thermal abuse for all variations.

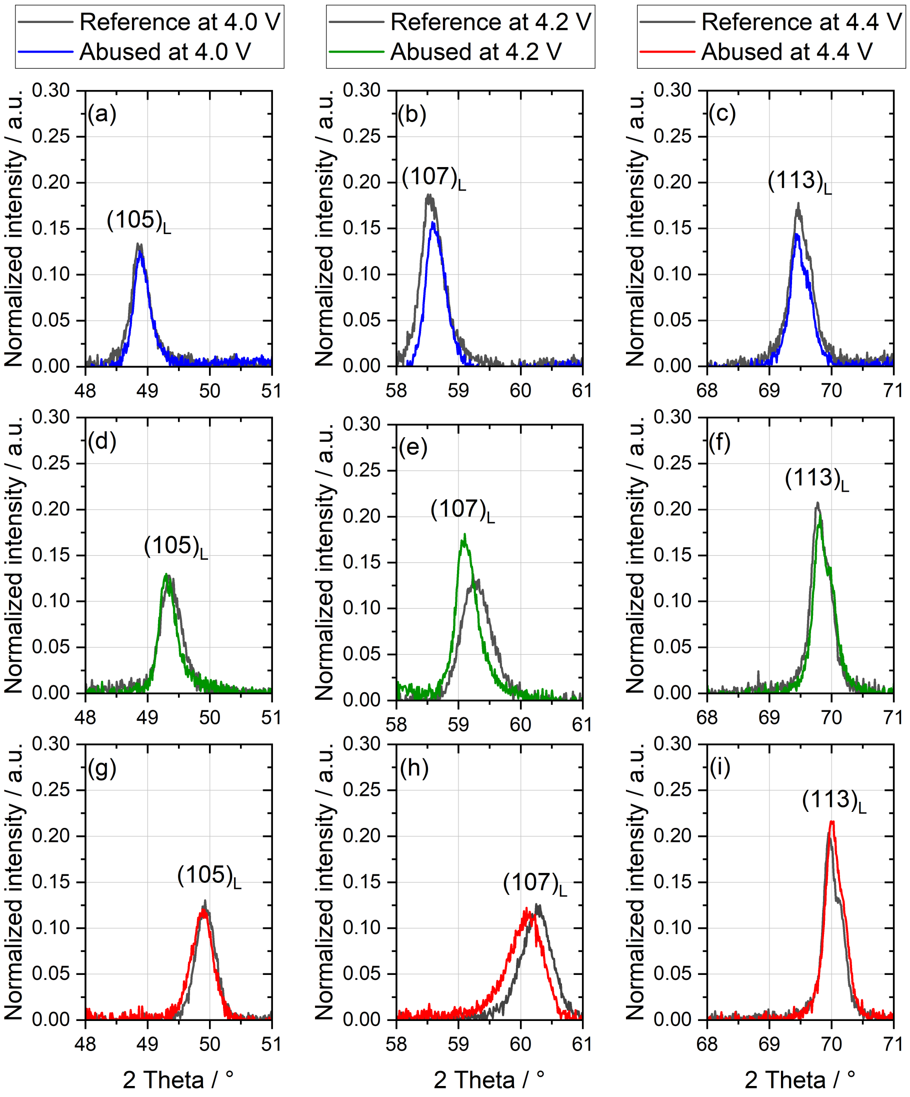

Fig. S7: XRD spectra reflections (105), (107) and (113) of NMC811 of cells with 300 ppm D_2O dosage and at OCPs (a)-(c) 4.0 V, (d)-(f) 4.2 V, and (g)-(i) 4.4 V before and after thermal abuse. Peaks were indexed to their corresponding reflection following Bak et al. [7]; L = layered oxide; S = spinel indicate their corresponding structure.

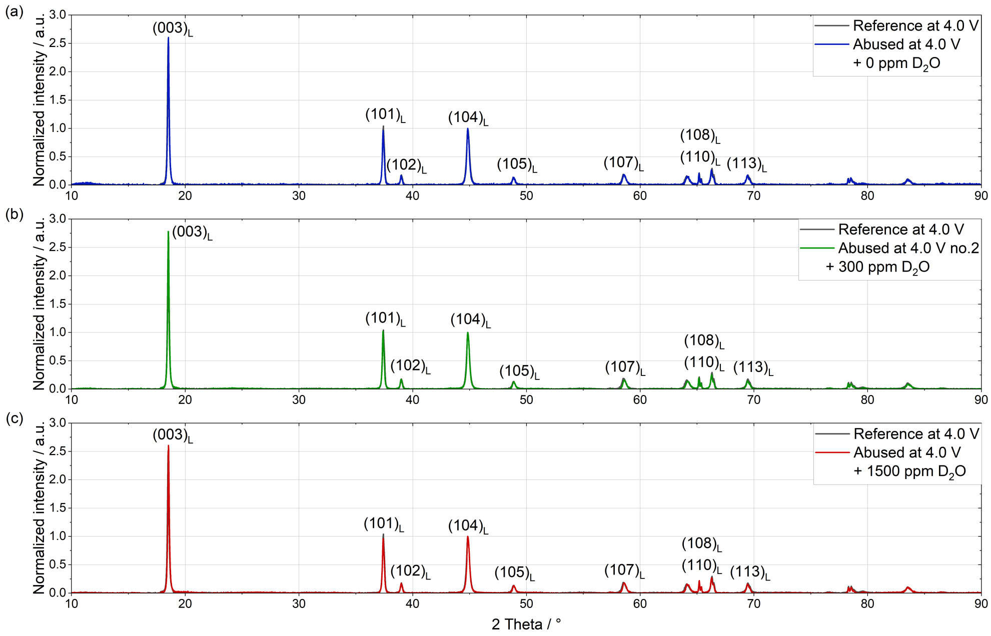

Fig. S8: XRD spectra from 2θ of 10° to 90° of NMC811 of cells at an OCP of 4.0 V and D_2O dosage of (a) 0 ppm, (b) 300 ppm, and (c) 1500 ppm before and after thermal abuse. Peaks were indexed to their corresponding reflection following Bak et al. [7]; L = layered oxide; S = spinel indicate their corresponding structure.

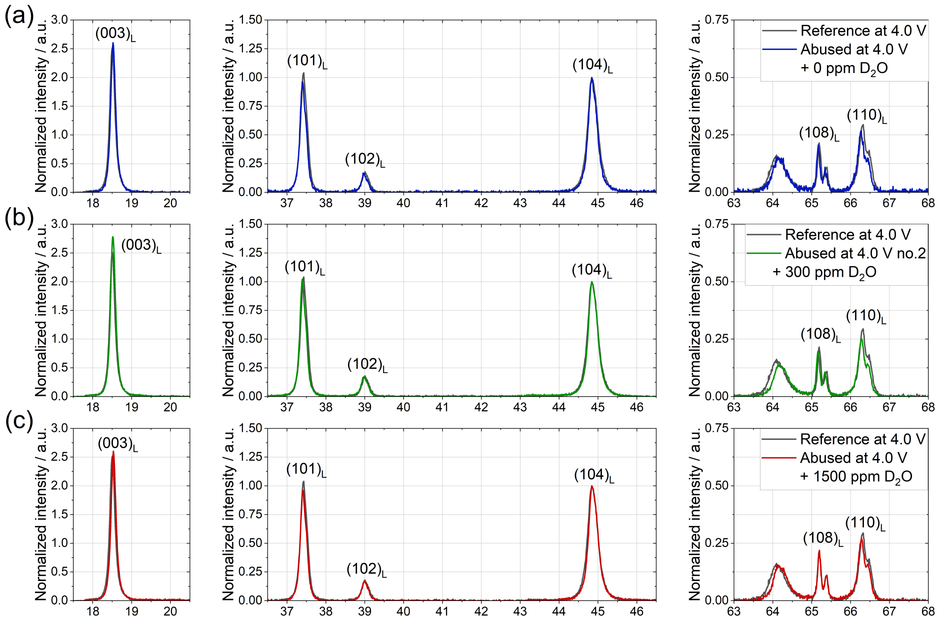

Fig. S9: Selected regions of XRD spectra of positive electrodes before and after thermal abuse at OCP of 4.0 V and with D_2O dosage of OCPs of (a) 0 ppm, (b) 300 ppm, and (c) 1500 ppm. Spectra were normalized to the height of (104) diffraction peak. Peaks were indexed to their corresponding reflection following Bak et al. [7]; L = layered oxide; S = spinel indicate their corresponding structure.

2.3. Scanning Electron Microscopy

In this section additional images of the SEM investigations are provided. Fig. S10 shows a selection of SEM images of positive and negative electrodes, and tab. S2 summarizes the information which experiments were performed before pictures were taken.

Letter	Electrode	D_2O Conc.	Voltage	Magnification	Additional Information
(a)	Negative	-	-	5000x	Pristine
(b)	Positive	-	-	5000x	Pristine
(c)	Negative	0 ppm	4.0 V	5000x	After formation
(d)	Positive	0 ppm	4.0 V	5000x	After formation
(e)	Positive	300 ppm	4.0 V	5000x	After heating
(f)	Positive	300 ppm	4.2 V	5000x	After heating
(g)	Positive	300 ppm	4.4 V	5000x	After heating
(h)	Negative	1500 ppm	4.0 V	250x	After heating
(i)	Negative	300 ppm	4.2 V	5000x	After heating
(j)	Negative	300 ppm	4.0 V	1000x	After heating
(k)	Negative	300 ppm	4.2 V	1000x	After heating
(l)	Negative	300 ppm	4.4 V	1000x	After heating

Tab. S2: Additional information to the SEM images shown in Fig. S10.

Pristine surfaces as well as surfaces after formation for both electrodes are included in Fig. S10 (a)-(d).

Additionally, the surfaces of the positive electrodes after thermal abuse with 300 ppm D_2O and (e) 4.0 V, (f) 4.2 V, and (g) 4.4 V are shown. No significant differences in surface film formation were found for the OCP variations: all samples show a similar extend of a surface film on the NMC primary particles. Additionally, during HPLC measurements, we observed only small changes in the amounts of carboxylic acids on the surfaces (compare Fig. S11). As described in the main text, we believe that the process of stepwise EC oxidation results in the formation of this surface film. This process is mainly limited by the concentration of water during thermal abuse and therefore does not result in significant changes in the surface film during the OCP variation.

In Fig. S10 (h), the surface of the negative electrode after thermal abuse with 1500 ppm D_2O is shown. This image a larger window of the surface of the negative electrode, revealing the extent of the polymer surface film observed. Red squares were added, marking areas of extensive bubble formation, indicating trapping of gases formed; we assume that these are formed during the thermal abuse. They are unlikely to inflate during the experiment; in the SEM chamber the bubbles inflate under the vacuum needed for the operation of the machine. This observation very likely has an effect on the HT-OEMS observation since the trapped gases cannot be detected.

Fig. S10 (i) shows the surface of the negative electrode after thermal abuse at 4.2 V. Images of 4.0 V and 4.4 V are included in the main text. Additionally, the surfaces less magnified of (j) 4.0 V, (k) 4.2 V, and (l) 4.4 V are shown. All samples of the OCP variation show different visual properties of the negative electrodes surface films. For 4.0 V and 4.2 V, the polymer described before fully covers multiple particles of the sur-

face. However, significant changes are found for 4.2 V; the film shows cracks, probably indicating less elasticity of the polymer. For 4.4 V, no polymer film can be found.

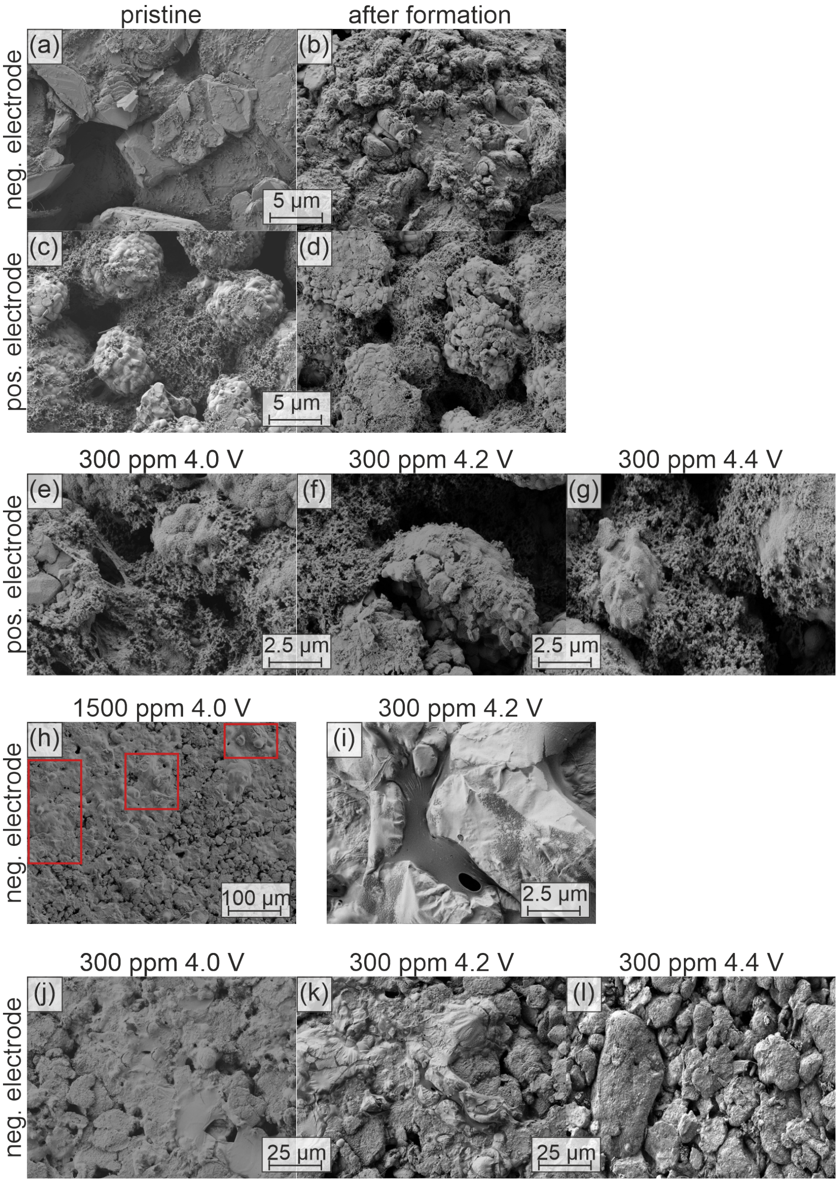

Fig. S10: Post-mortem SEM images of the electrode surfaces: Surface of negative electrode in (a) pristine state and (b) after formation. Surface of positive electrode in (c) pristine state and (d) after formation. Surface of positive electrodes after thermal abuse with 300 ppm dosage and OCPs of (e) 4.0 V, (f) 4.2 V, and (g) 4.4 V. Surface of negative electrodes after thermal abuse with 1500 ppm dosage and an OCP of (h) 4.0 V and with 300 ppm dosage and an OCP of (i) 4.2 V. Surface of negative electrodes after thermal abuse with 300 ppm dosage and OCPs of (j) 4.0 V, (k) 4.2 V, and (l) 4.4 V. See tab. S2 for magnification and electrode information.

2.4. High performance liquid chromatography

The results of the HPLC measurements are shown in Fig. S11 and discussed in the main text.

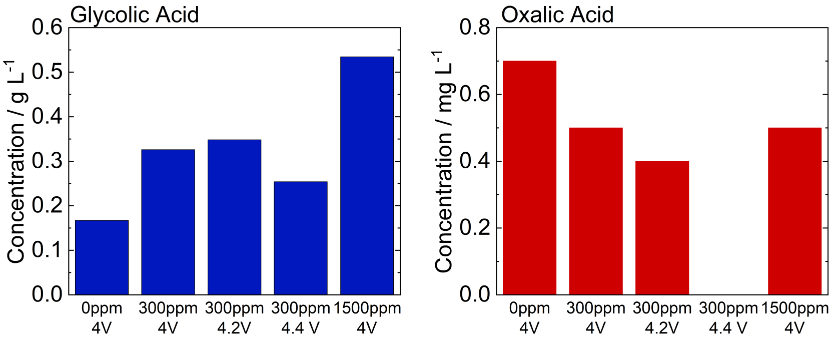

Fig. S11: Amounts of glycolic acid and oxalic acid found in HPLC, from submerging positive electrode in H_2O after thermal abuse.

2.5. Description of the control experiment for ethylene carbonate ring-opening and ethylene glycol oxidation

Two control experiments (CE) were performed to validate the degradation of EC to EG (CE No.1) and to compare the reaction kinetics of the oxidation of EC/DMC electrolyte and EG on delithiated NMC (CE No.2).

For CE No.1, we mixed 1.69 g of EC, 0.35 g of H_2O and 5 mg of LiF in a 20 mL headspace vial and sealed it under argon atmosphere. The vial was heated to 80 °C (roughly CO_2 gassing onset in HT-OEMS) and held at that temperature for 2 h. Gases were extracted from vial headspace with a syringe and analyzed in GC, confirming the presence of CO_2. Additionally, liquid residuals in the vial were analyzed in HPLC, confirming the presence of EG.

Gaseous products were analyzed with a GC (GC-2030, Shimadzu). The detector used was a BID, with a temperature set at 250 °C and a gas flow of 50 mL min^{-1} using He as a carrier gas. GC calibration was performed using two different CO_2 concentrations from 0 vol% to 0.5 vol%. Evaluation of the measurements was performed using Shimadzu's PostRun Analysis Software.

For CE No.2, cells with the same chemistry, but higher mass loading of 3.5 mg cm^{-2}, were cycled with the same formation protocol as previous and charged to 4.0 V. Subsequently, cells were disassembled inside a glovebox and the positive electrodes were transferred into HT-OEMS cells with a new PTFE separator. Cells contained the delithiated NMC with either 100 μL of standard electrolyte (1M $LiPF_6$ in EC/DMC (50/50)) or 50 μL of ethylene glycol. Cells were connected to HT-OEMS setup, flushed for 10 min with 12 mL min^{-1} Argon and subjected to thermal abuse with the same profile as full cell experiments (2 °C min^{-1} from room temperature to 125 °C). Subsequently, the cells were disassembled in the glovebox and the positive electrodes were washed in DMC and analyzed in HPLC.

The observed peak areas of glycolic acid, oxalic acid and acetic acid are summarized in tab. S3.

Tab. S3: Peak area in mAu s observed in HPLC of glycolic acid, oxalic acid, and acetic acid after rinsing of positive electrode. Samples are NMC811 with 1M $LiPF_6$ in EC/DMC and ethylene glycol after thermal abuse (control experiment No.2).

Electrolyte	Peak area of glycolic acid / mAu s^{-1}	Peak area of oxalic acid / mAu s^{-1}	Peak area of acetic acid / mAu s^{-1}
1M $LiPF_6$ in EC/DMC	0.27	0.16	0.12
EG	0.30	2.28	0.61

2.6. Detailed reaction mechanism for EC degradation at NMC electrodes

For R.1 to R.4 in Fig. 6, previous studies have provided mechanistic insights into reaction steps shown in Fig. S12 [6, 8, 9]. The proposed reaction steps, including intermediate states, can be used for future DFT calculations to further confirm the feasibility of these reactions. Rinkel et al. [6] suggested that, when EC is directly oxidized by oxygen (R.1), one of the CH_2 groups in the ring undergoes hydrogen abstraction, forming a hydroperoxide intermediate (R.1.1). The oxygen atom that is not directly bonded to the carbon then abstracts the other hydrogen from the carbon to form water (R.1.2 and R.1.3), which triggers formation of a carbonyl group. We suggest that homolytic, rather than heterolytic, OO bond cleavage is favored for the peroxide intermediate for R.1.3, R.3.3 and R.4.3. Following the mechanism proposed by Jung et al. [8], the same three steps repeat at the other CH_2 group in the ring (R.1.7.1 and R.1.7.2), and the formed molecule decomposes to form two CO_2 and CO (R.1.3.2). Alternatively, the water formed in reaction step R.1.3 attacks the carbonyl group, triggering a ring-opening (R.1.5). In a final step, the molecule undergoes decarboxylation to form CO_2 and glycolic acid (R.1.6) - in total, six steps from EC to glycolic acid.
For reaction R.2, the mechanism proposed by Hu et al. [9] for EC and methanol was adjusted for EC and water and is shown, where LiF coordinates with the carbonate group of EC and H_2O (R.2.1). The water then attacks the carbonyl carbon of the carbonate group, leading to ring opening of the formation of hydroxyethyl monocarbonate. This intermediate subsequently undergoes decarboxylation to form CO_2 and EG. Since this pathway is catalyzed by dissolved LiF, the production of EG is not limited to e.g. the NMC surfaces, but can take place in the bulk electrolytes. R.3 and R.4 each proceed in three steps to oxidize EG and glycolic acid, respectively; they follow the same hydrogen abstraction steps as described for R.1.1, R.1.2, and R.1.3.

2.7. Proposed mechanism leading to Deuterium-Hydrogen swap during chemical oxidation

Fig. S13 shows the positions of hydrogen and deuterium during the hydrolysis and oxidation of EC. It is assumed here that the EC molecule only contains hydrogen and that species can not encounter hydrogen-deuterium exchanges, which might occur in the real system. During EC hydrolysis the ring is opened and CO_2 is released. D_2O splits, and two hydroxide groups are formed with Deuterium bonded to the oxygen, forming

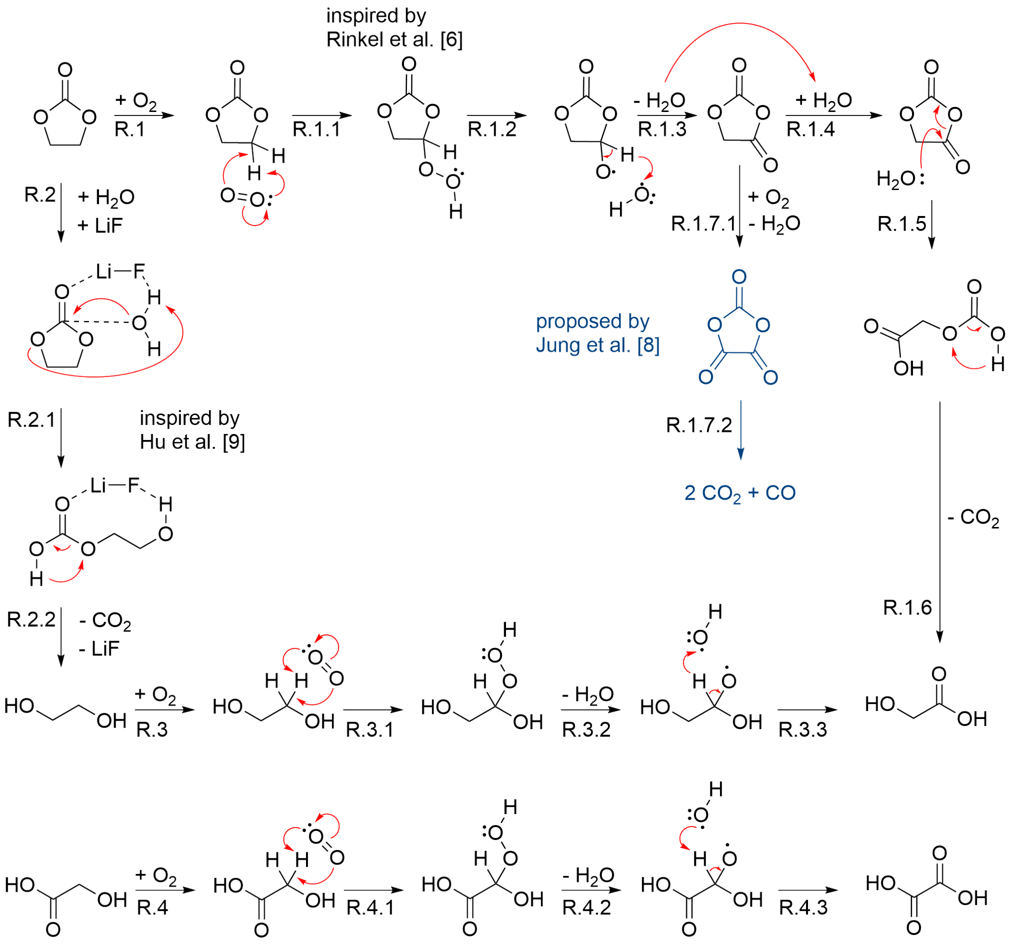

Fig. S12: Detailed mechanism including reaction intermediates of EC oxidation (R.1), EC to EG conversion (R.2), EG oxidation (R.3), and glycolic acid oxidation (R.4) from Fig. 6.

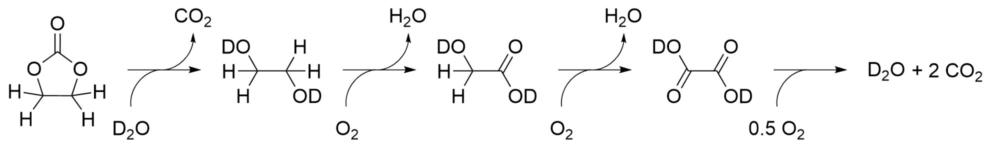

Fig. S13: Positions of hydrogen and deuterium during the hydrolysis and oxidation of EC with D_2O.

EG. Subsequently, first ethylene glycol is oxidized to glycolic acid and then glycolic acid to oxalic acid, both with oxygen from the NMC lattice. The reactions produce water from the remaining hydrogen atoms from the initial EC molecule, compare mechanism suggested by Rinkel et al. [6]. In the final step, for the full oxidation, D_2O is produced with two CO_2.

3. DFT calculations

3.1. Thermodynamic values of the DFT calculation with triplet oxygen

In literature discussions about the release of the state of oxygen leading to chemical oxidation are still ongoing [10, 11]. We included calculated values of ΔH_R, ΔS_R, and ΔG_R for oxidation of EC, EG and carboxylic acids with triplet oxygen at 25 °C and 126.85 °C.

Tab. S4: Gibbs free energy and reaction enthalpies for different pathways of oxidation of organic species in the electrolyte with triplet oxygen at 25 °C and 126.85 °C.

No.	Reaction	25 °C		126.85 °C	
		ΔG_R kJ mol^{-1}	ΔH_R kJ mol^{-1}	ΔG_R kJ mol^{-1}	ΔH_R kJ mol^{-1}
R1	EC + 2 O_2 → 2 CO_2 + 2 H_2O + CO	-900	-836	-922	-834
R2	EC + H_2O → CO_2 + EG	-11	-18	-8	-17
R3	EG + O_2 → H_2O + glycolic acid	-417	-417	-416	-417
R4	glycolic acid + O_2 → H_2O + oxalic acid	-382	-382	-382	-382
R5	oxalic acid + 0.5 O_2 → H_2O + 2 CO_2	-346	-314	-358	-313
R6	EG + 0.5 O_2 → acetic acid + H_2O	-319	-297	-327	-296
R7	EC + 1.5 O_2 → CO_2 + 2 H_2O + 2 CO	-644	-541	-679	-538

References

[1] L. Schmidt, K. Hankins, L. Bläubaum, M. Gerasimov, U. Krewer, *Chemical Science* **2025**, *16*, 5118–5128.

[2] M. Stich, M. Göttlinger, M. Kurniawan, U. Schmidt, A. Bund, *The Journal of Physical Chemistry C* **2018**, *122*, 8836–8842.

[3] E. W. C. Spotte-Smith, T. B. Petrocelli, H. D. Patel, S. M. Blau, K. A. Persson, *ACS Energy Letters* **2023**, *8*, 347–355.

[4] L. Hartmann, L. Reuter, L. Wallisch, A. Beiersdorfer, A. Adam, D. Goldbach, T. Teufl, P. Lamp, H. A. Gasteiger, J. Wandt, *Journal of The Electrochemical Society* **2024**, *171*, 060506.

[5] H. Zhang, J. Xue, Y. Qin, J. Chen, J. Wang, X. Yu, B. Zhang, Y. Zou, Y.-h. Hong, Z. Li, Y. Qiao, S.-G. Sun, *Small* **2024**, *20*, 2406110.

[6] B. L. D. Rinkel, D. S. Hall, I. Temprano, C. P. Grey, *Journal of the American Chemical Society* **2020**, *142*, 15058–15074.

[7] S.-M. Bak, E. Hu, Y. Zhou, X. Yu, S. D. Senanayake, S.-J. Cho, K.-B. Kim, K. Y. Chung, X.-Q. Yang, K.-W. Nam, *ACS Applied Materials & Interfaces* **2014**, *6*, 22594–22601.

[8] R. Jung, M. Metzger, F. Maglia, C. Stinner, H. A. Gasteiger, *Journal of The Electrochemical Society* **2017**, *164*, A1361–A1377.

[9] H. Hu, C. Luo, B. Wang, T. Lai, G. Zhang, G. Gao, *Molecular Catalysis* **2023**, *538*, 113010.

[10] J. Wandt, A. T. Freiberg, A. Ogrodnik, H. A. Gasteiger, *Materials Today* **2018**, *21*, 825–833.

[11] R. C. McNulty, K. D. Jones, B. M. G. Denison, E. Hampson, I. Temprano, D. A. Walsh, H. W. Lam, G. N. Newton, W. M. Dose, C. P. Grey, L. R. Johnson, *Energy & Environmental Science* **2025**, *18*, 7603–7609.
