# Supplementary figures and images for: High-temperature chemical oxidation pathways in lithium-ion batteries: mechanistic insights into ethylene carbonate decomposition
Source: Chem Sci. 2026 Mar 9;17(18):9049–60. doi: 10.1039/d6sc00426a (PMC12990432; doi:10.1039/d6sc00426a)

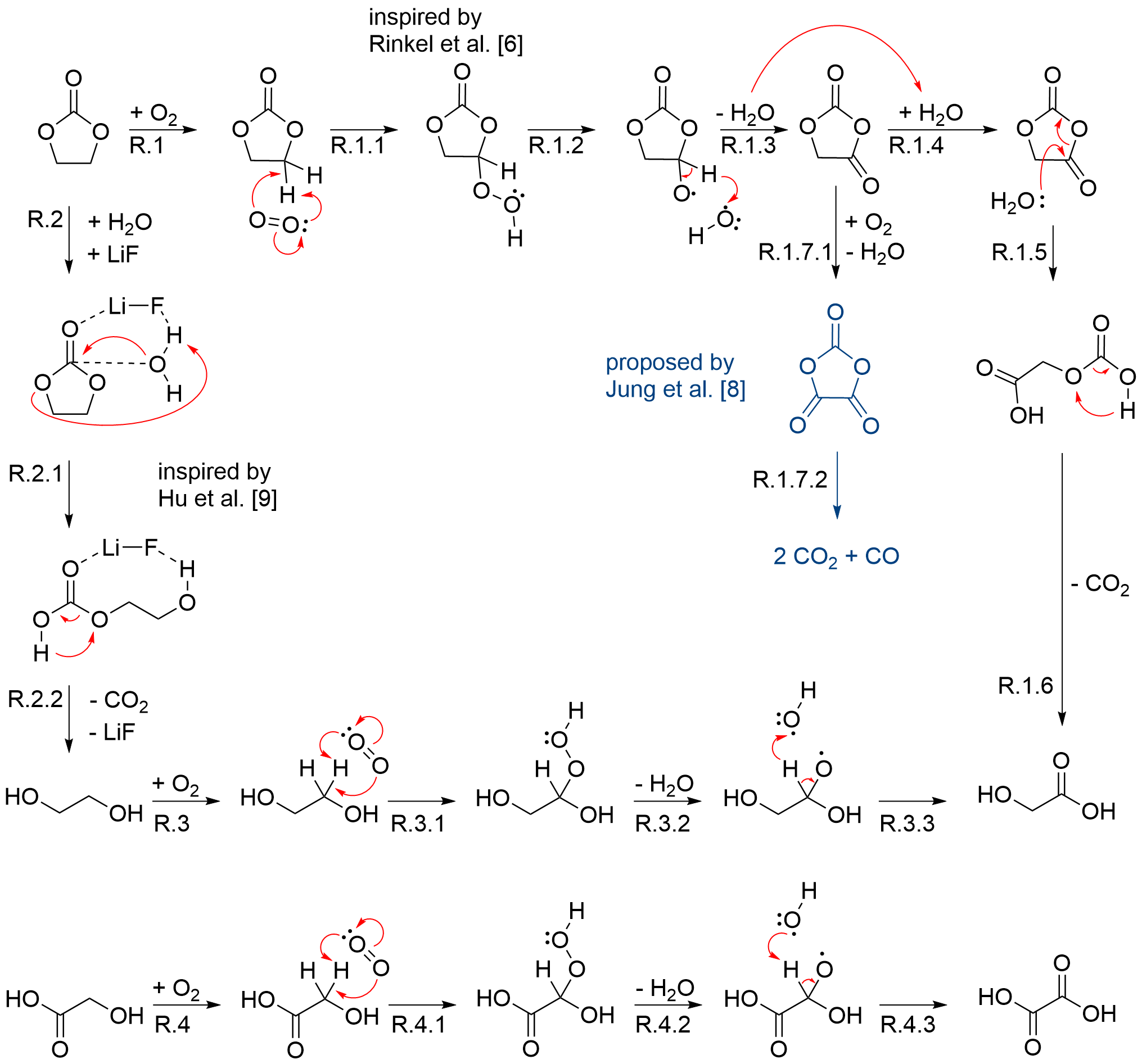

Supplement: SC-017-D6SC00426A-s002 [file SC-017-D6SC00426A-s002.zip › SI/251022_detailed_mechanism_EC_EG_oxidation.png]

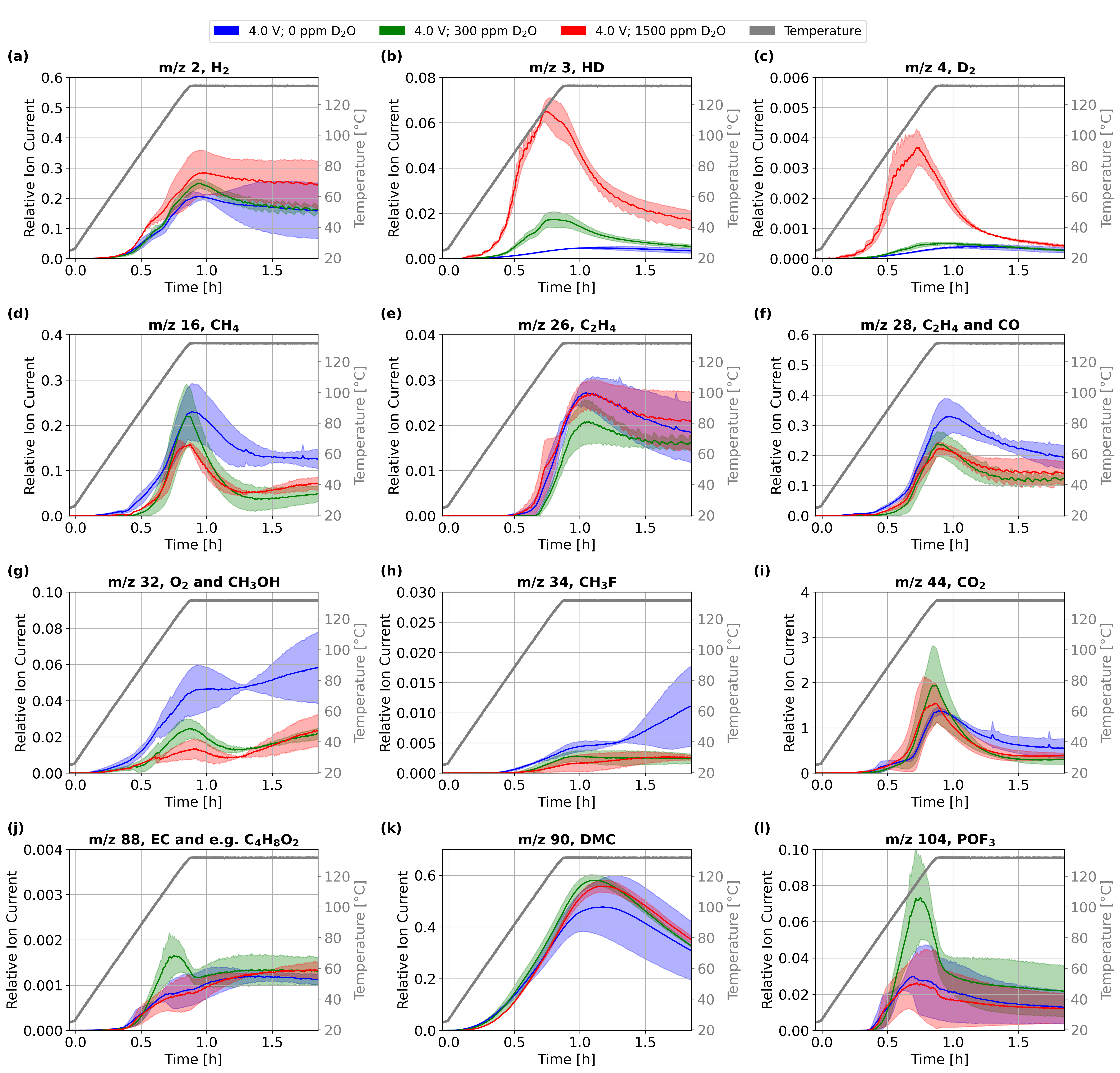

Supplement: SC-017-D6SC00426A-s002 [file SC-017-D6SC00426A-s002.zip › SI/D2O_SI.png]

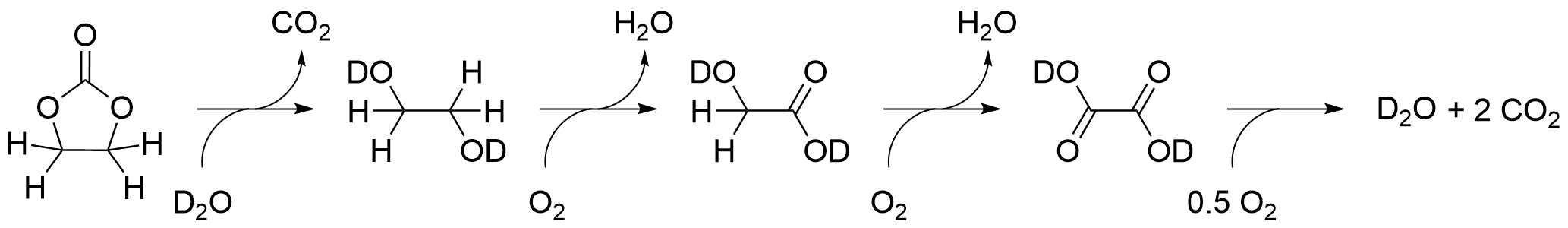

Supplement: SC-017-D6SC00426A-s002 [file SC-017-D6SC00426A-s002.zip › SI/deuterium_during_oxidation.png]

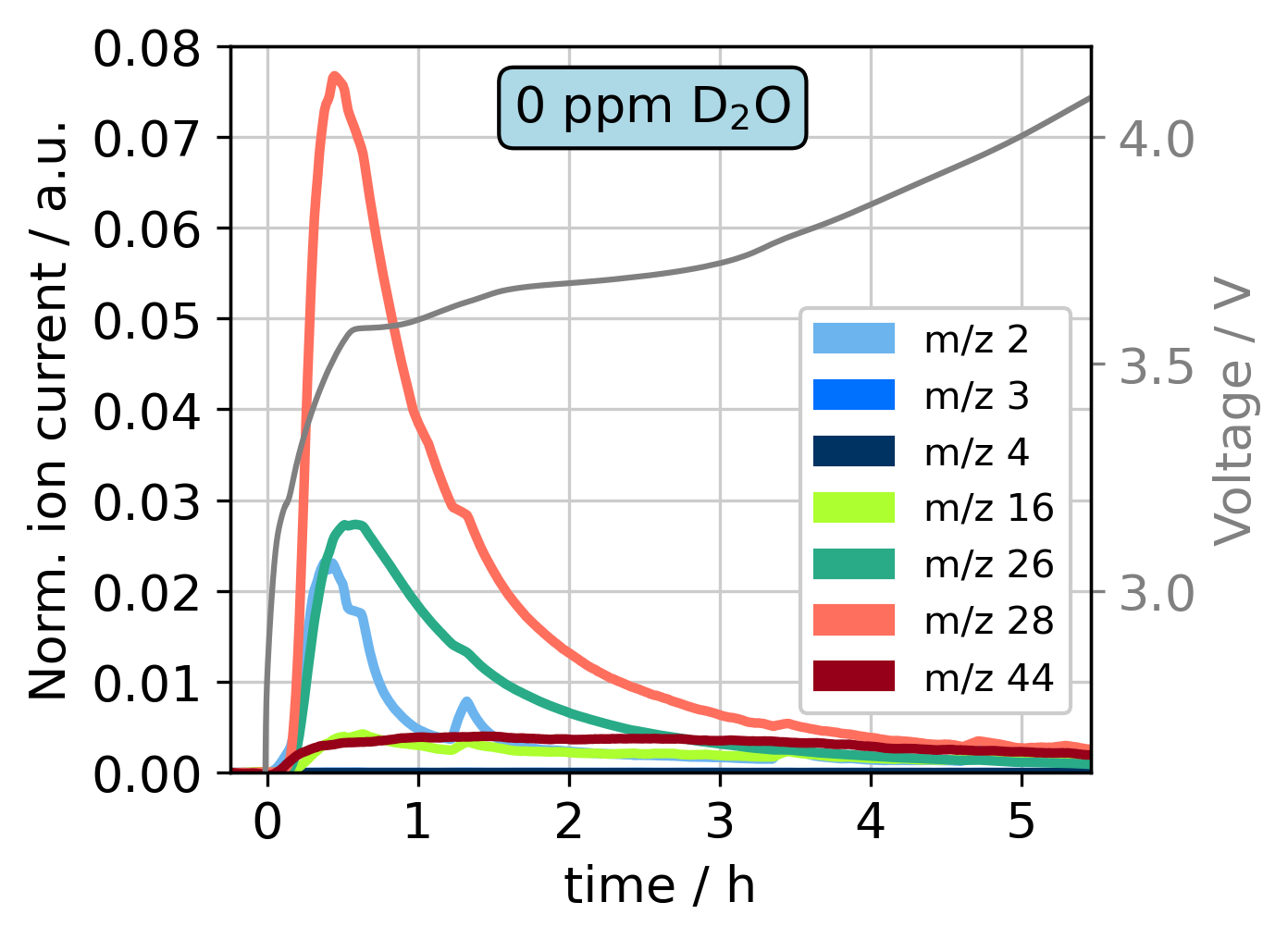

Supplement: SC-017-D6SC00426A-s002 [file SC-017-D6SC00426A-s002.zip › SI/formation442.png]

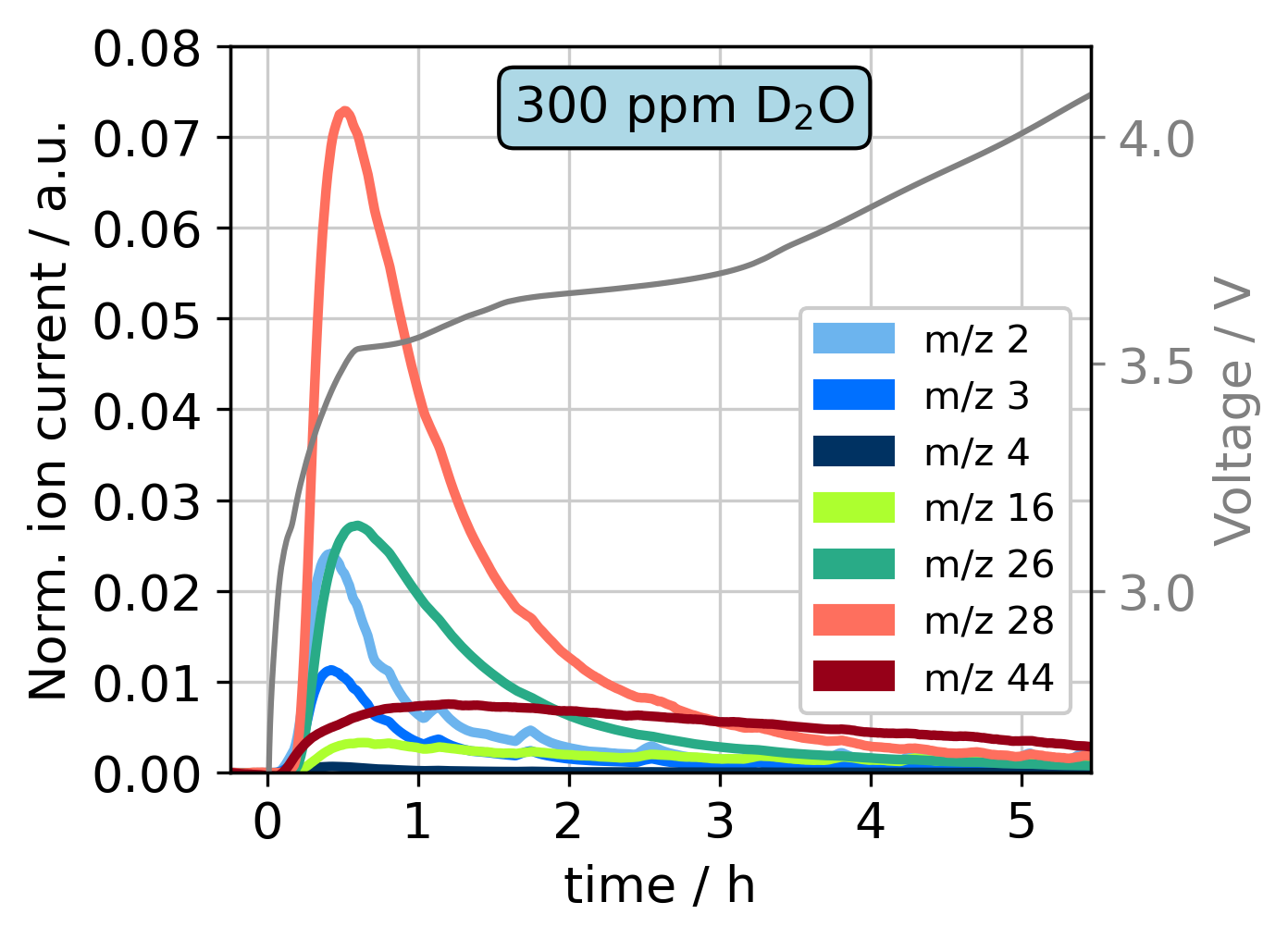

Supplement: SC-017-D6SC00426A-s002 [file SC-017-D6SC00426A-s002.zip › SI/formation467.png]

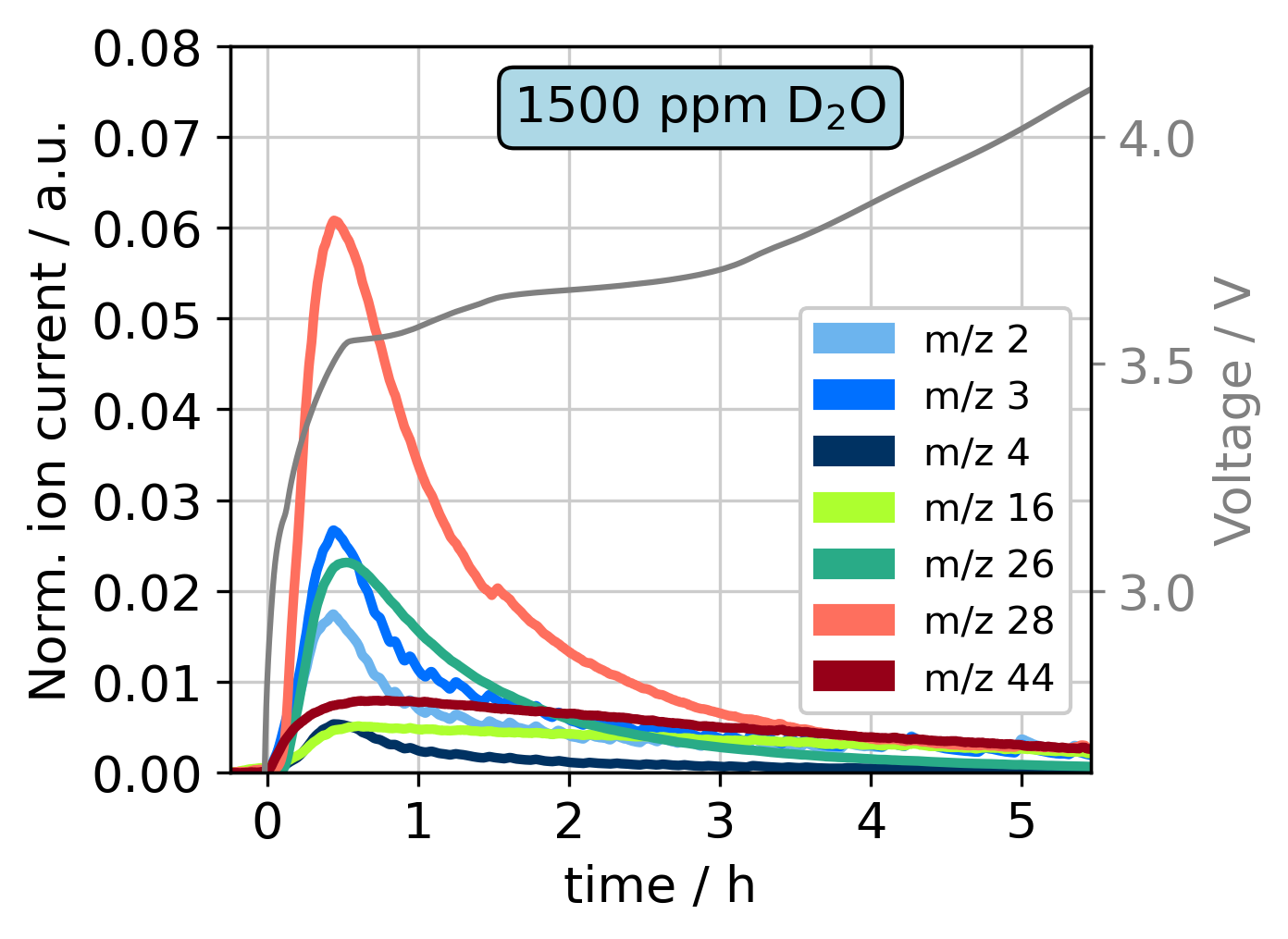

Supplement: SC-017-D6SC00426A-s002 [file SC-017-D6SC00426A-s002.zip › SI/formation503.png]

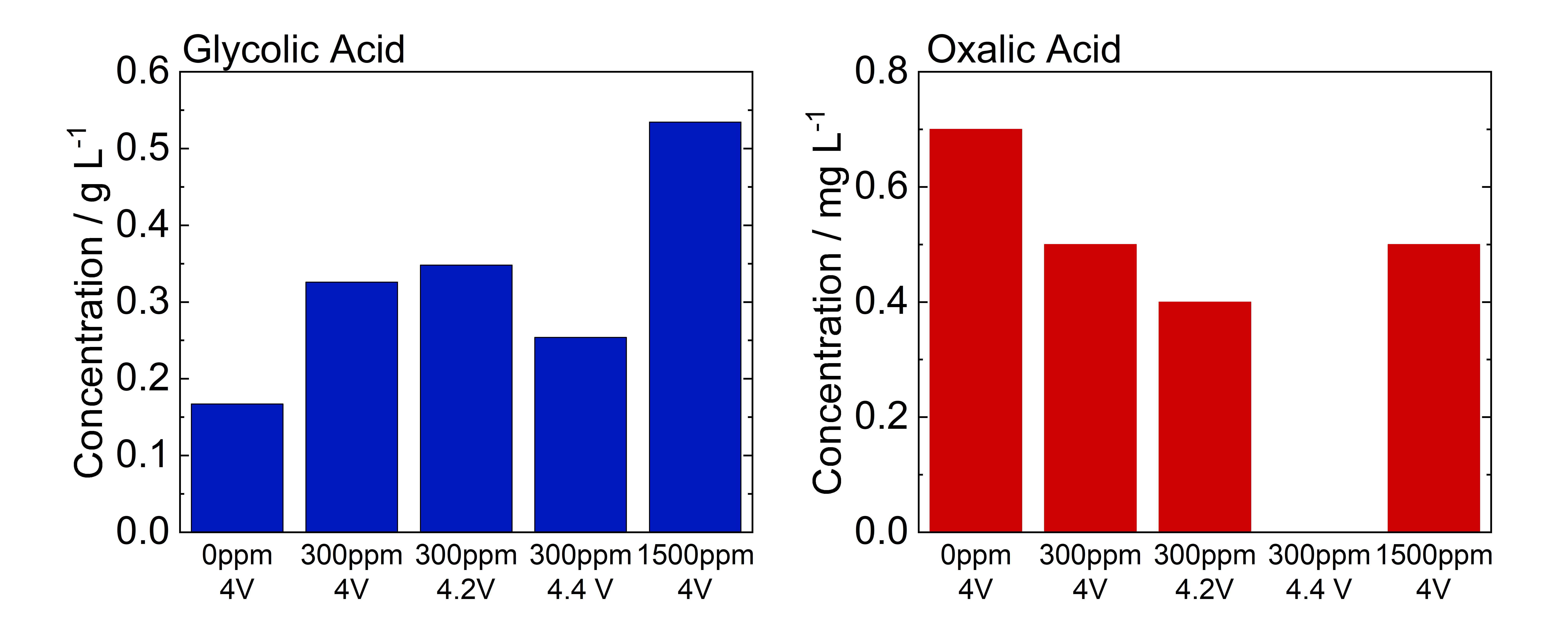

Supplement: SC-017-D6SC00426A-s002 [file SC-017-D6SC00426A-s002.zip › SI/HPLC_acids.png]

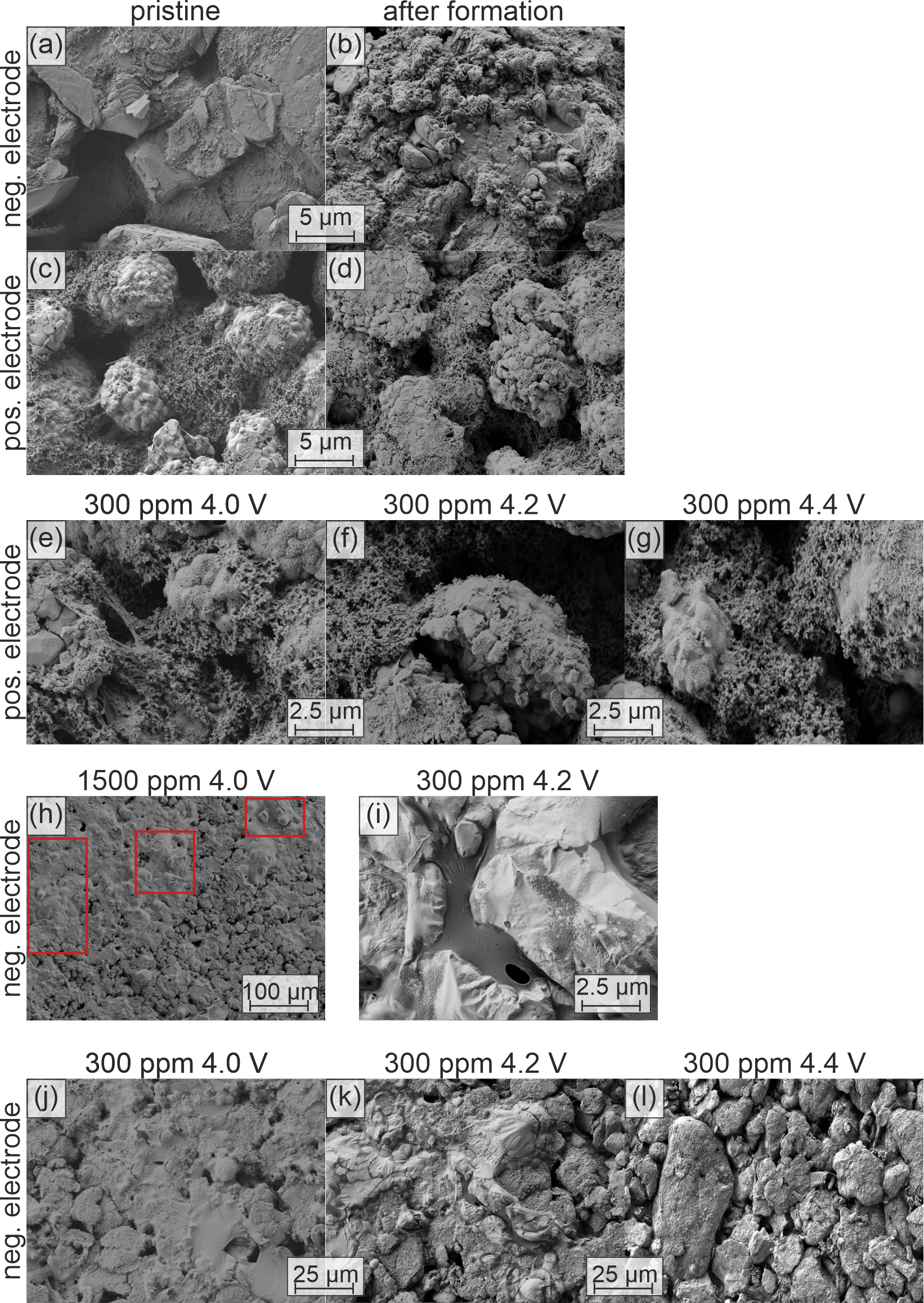

Supplement: SC-017-D6SC00426A-s002 [file SC-017-D6SC00426A-s002.zip › SI/SI_SEM.png]

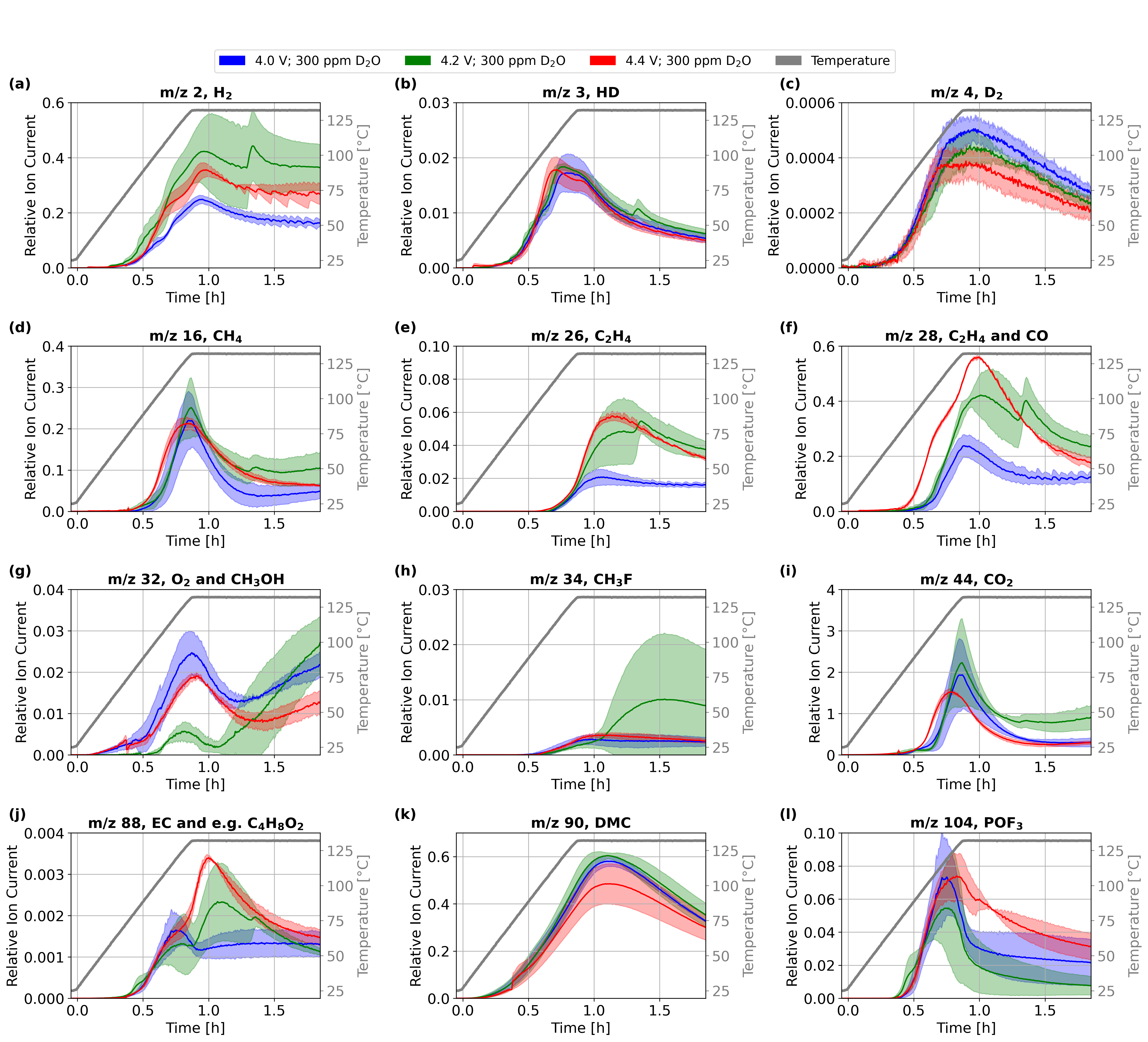

Supplement: SC-017-D6SC00426A-s002 [file SC-017-D6SC00426A-s002.zip › SI/SOCs_SI.png]

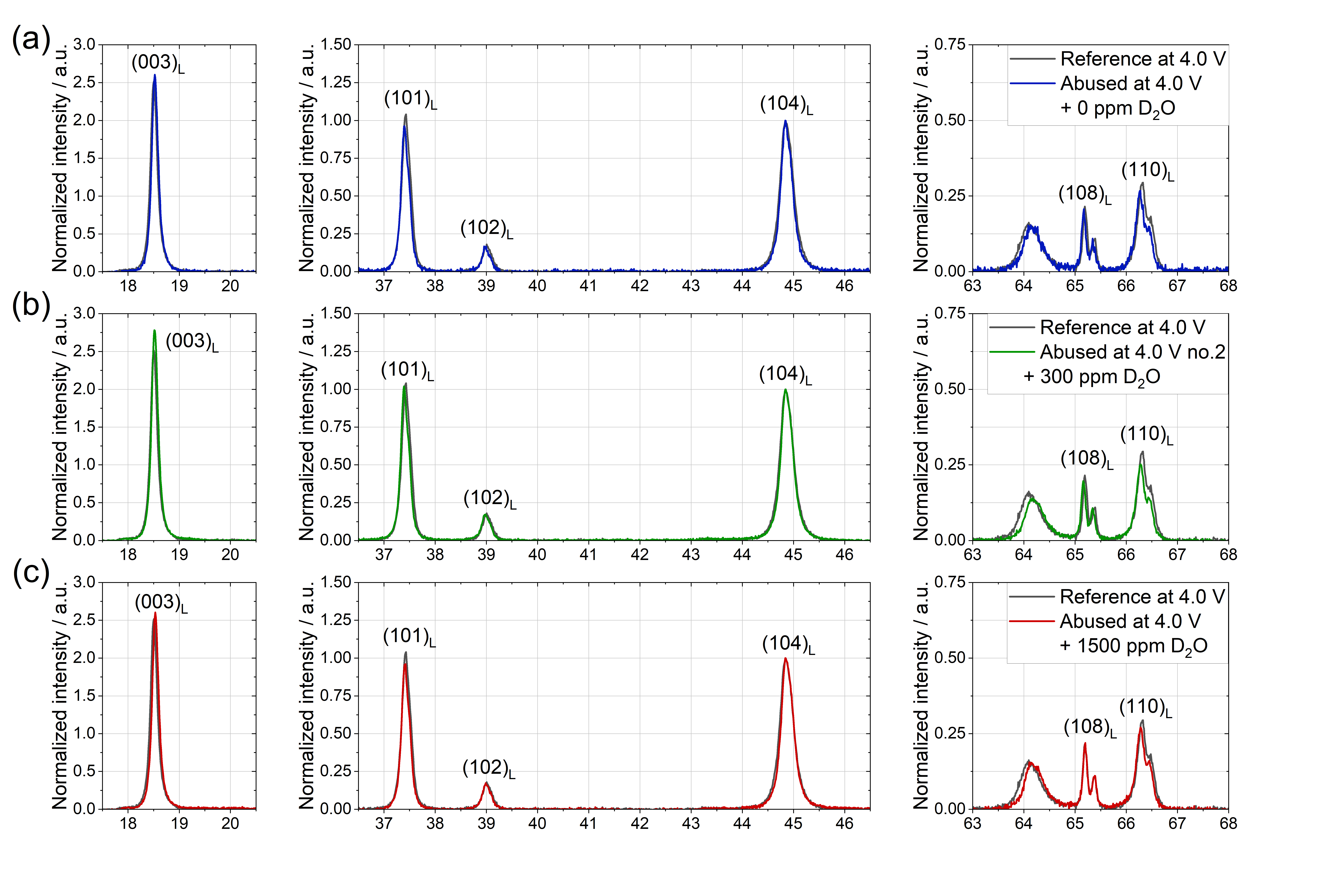

Supplement: SC-017-D6SC00426A-s002 [file SC-017-D6SC00426A-s002.zip › SI/XRD_D2O.png]

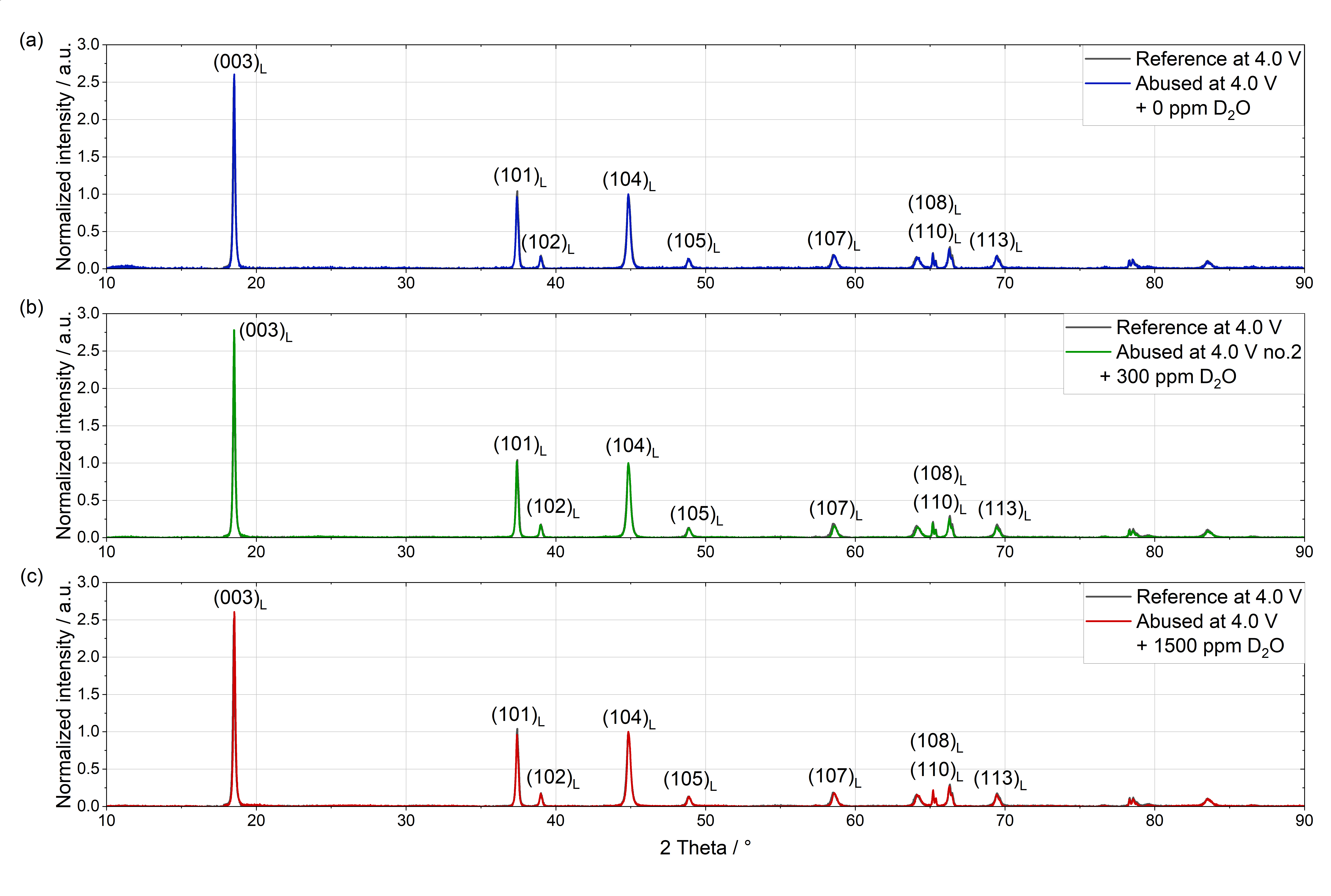

Supplement: SC-017-D6SC00426A-s002 [file SC-017-D6SC00426A-s002.zip › SI/XRD_D2O_full.png]

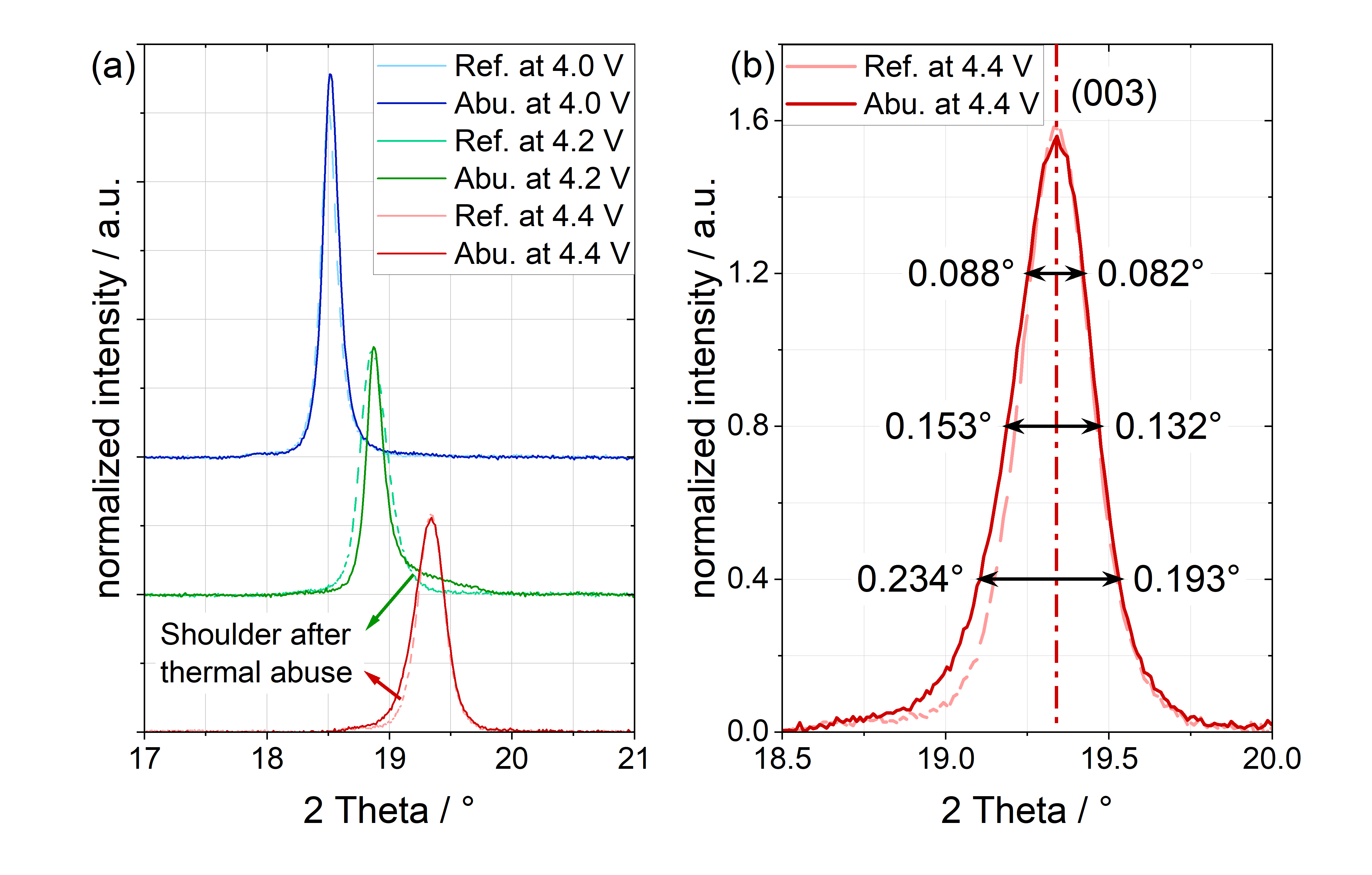

Supplement: SC-017-D6SC00426A-s002 [file SC-017-D6SC00426A-s002.zip › SI/XRD_SOC_asymmetry.png]

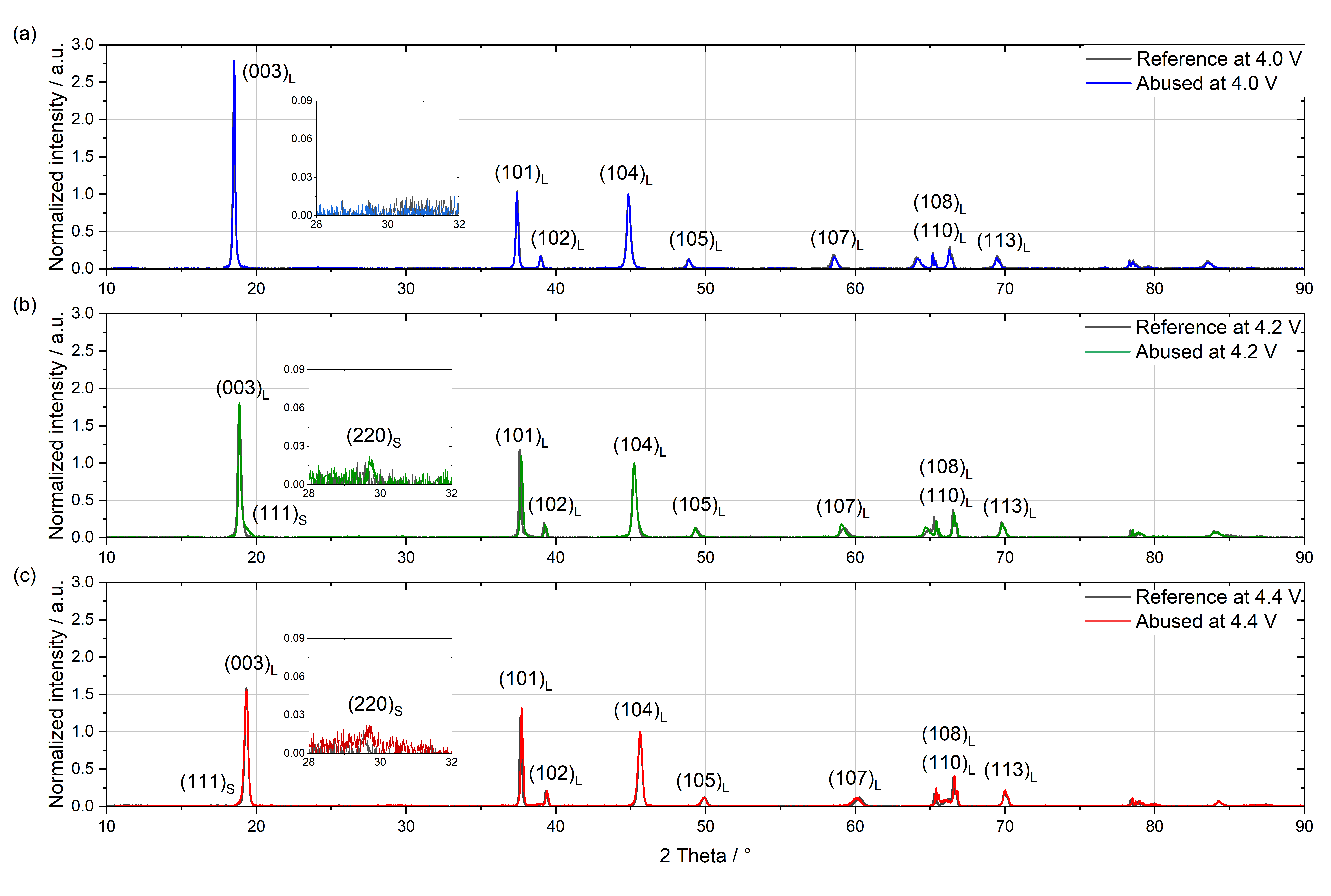

Supplement: SC-017-D6SC00426A-s002 [file SC-017-D6SC00426A-s002.zip › SI/XRD_SOC_full.png]

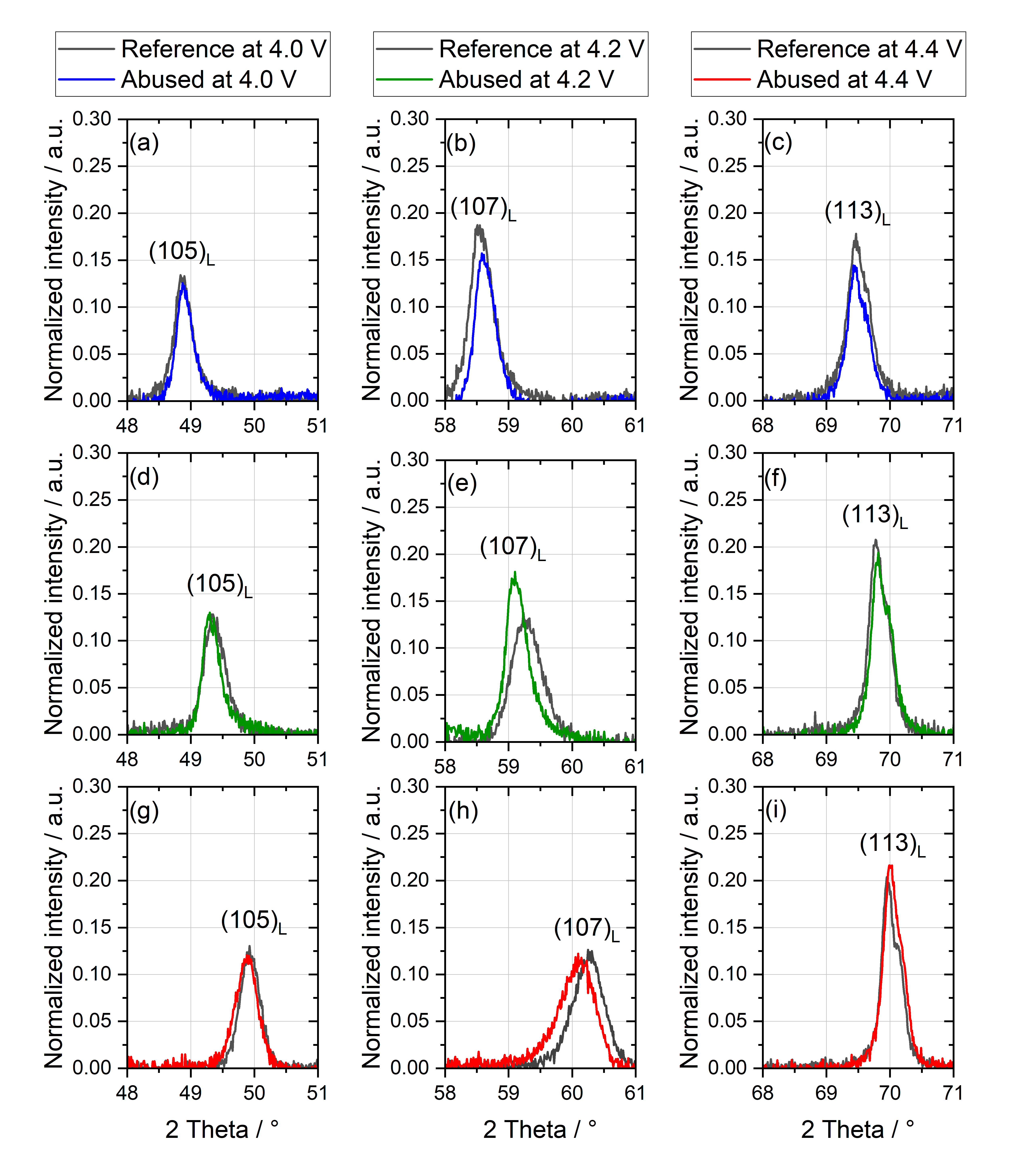

Supplement: SC-017-D6SC00426A-s002 [file SC-017-D6SC00426A-s002.zip › SI/XRD_SOC_SI.png]

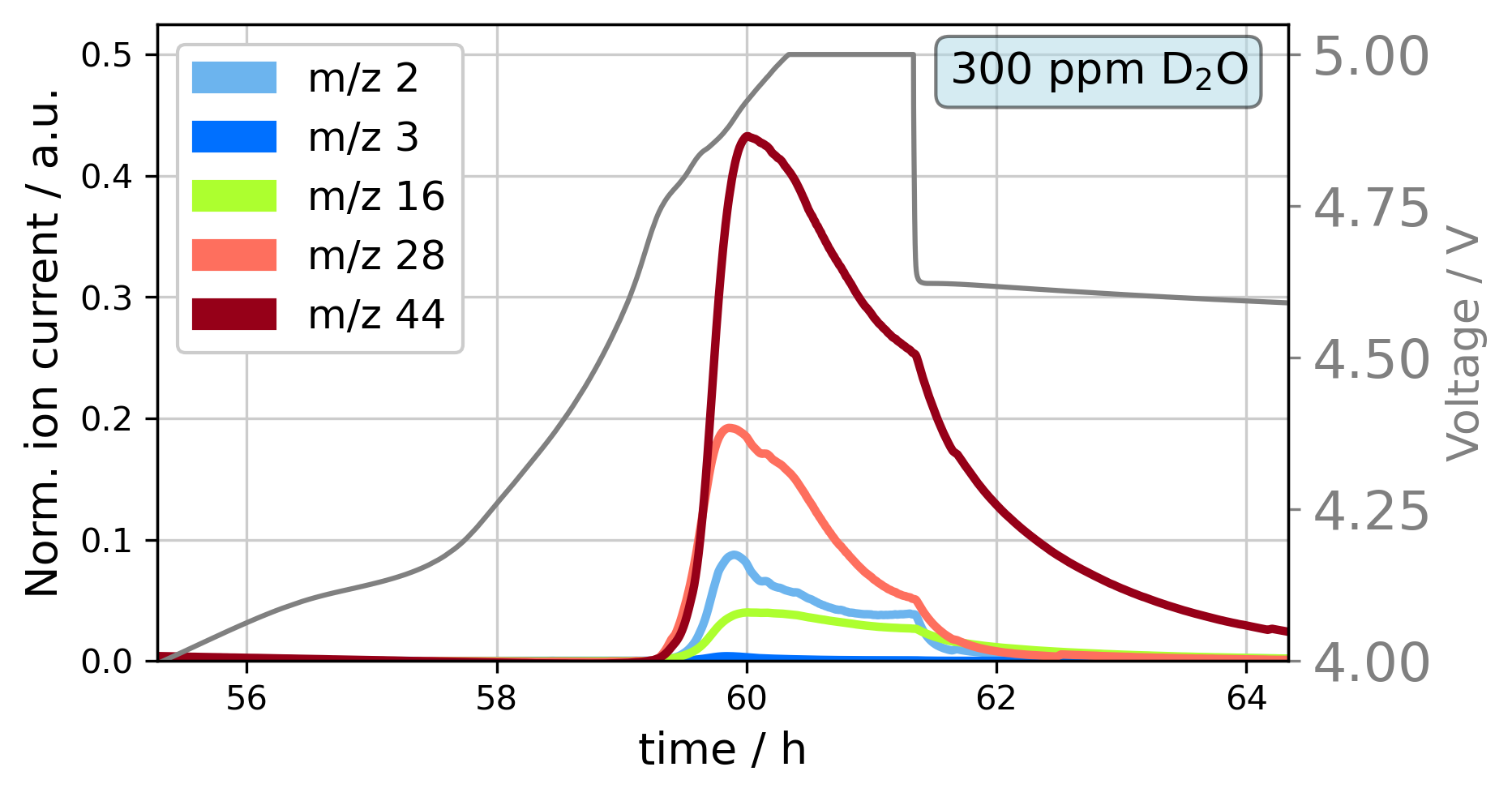

Supplement: SC-017-D6SC00426A-s002 [file SC-017-D6SC00426A-s002.zip › SI/zelle482.png]
